# Supplementary material for: Ultrafast photochemistry produces superbright short-wave infrared dots for low-dose in vivo imaging
Source: Nat Commun. 2020 Jun 10;11:2933. doi: 10.1038/s41467-020-16333-2 (PMC7286912; doi:10.1038/s41467-020-16333-2)
Supplement: Supplementary file 1 — Supplementary Information [file 41467_2020_16333_MOESM1_ESM.pdf]

Supplementary Information for

Ultrafast photochemistry produces superbright short-wave infrared dots for low-dose in vivo imaging

Harrisson D. A. Santos et al.

Supplementary Methods

Study of in vivo *thermal loading*

The magnitude of the laser-induced thermal loading during *in vivo* imaging experiments was analysed in an anesthetized mouse. An infrared thermographic camera (FLIR E-40) was used to measure the surface temperature of the mouse under 14 different irradiation power densities: 0.3, 5, 20, 40, 50, 70, 90, 112, 130, 150, 173, 200, 227 and 240 mW cm^{-2}. The temperature was recorded after 1 min of 808 nm laser irradiation at each power density.

Subchronic toxicological studies

To test the long-term biocompatibility of Ag_2S superdots, we performed a 28-days subchronic toxicological experiment complemented with histological analyses on splenic, hepatic, and renal tissue samples. CD1 mouse strain is optimal to conduct toxicological studies due to their increased fidelity to represent the genetic heterogeneity background of a population compared to other typical outbred strains (such as C57/Bl6). Nineteen 1-2 month-old female CD1 mice under 2% of isofluorane-induced anaesthesia were intravenously injected through the retro-orbital sinus with a 100 µl of a dispersion at a concentration of 0.15 mg mL^{-1} solution of Ag_2S superdots in sterile-PBS. This injection volume is identical to that used in our work for *in vivo* imaging. As a control, mice were injected with the same volume of PBS. Five groups (n = 3) were injected with Ag_2S and euthanized 24 h, 7 days, 14 days, 21 days and 28 days post-injection. To include an indicative control of both potential early acute and late sub-chronic stages, two additional groups (n = 2) were injected with PBS and euthanized after 24 h and 28 days respectively. We chose to reduce the number of animals in these groups and to limit the study to the acute and last stage after injection to minimize the number of animals used in the study. Mice previously anesthetized by isoflurane (5%) were euthanized by beheading, the blood (1-1.2 mL) was collected and serum was obtained after coagulation and centrifugation (1 hours at room temperature and 2 hours at 4 °C followed by 15 minutes of 10,000g centrifugation at 4 °C). All samples were stored at -80 °C and biochemical determinations of colorimetric endpoint and 2 point kinetic curves assays were performed by in collaboration with a laboratory of clinical analysis (Laboratorio Alemany, Madrid, Spain). Toxicological assays were performed to ascertain possible changes in hepatic (ALT-Alanine transaminase, ALP-Alkaline phosphatase, and AST-Aspartate transaminase), renal (creatinine) and hemolytic profiles (total bilirubin), and a toxicokinetic profile was obtained for Ag_2S treated mice and euthanized at increasing time points up to 28 days.

Cytotoxicity

We have performed *in vitro* cytotoxicity assays in HeLa (human cervical adenocarcinoma), NIH-3T3 (swiss mouse embryo fibroblasts) and IMR-90 (human lung fibroblasts) cells using the thiazolyl blue tetrazolium bromide (3-(4,5-dimethyl-thiazol-2yl)-2,5-diphenyltetrazolium bromide, MTT) assay. Briefly, cells in complete medium (DMEM with 10 % FCS, pyruvate, glutamine, penicillin-streptomycin and gentamicin) were seeded in 96-well culture plates at a density of 10^4 cells/well. After 24 h of incubation (37 ºC, 5 % CO_2), Ag_2S superdots were added to cells at different concentrations up to 200 mg/mL and further incubated for 48 h. Then, MTT solution was added for 4 h to the plate and afterwards, the MTT reaction was stopped by adding a solution of dimethylformamide-SDS. Finally, the plate was gently shaken for 2 h to dissolve formazan crystals prior to measuring 570/590 nm absorbance in an Appliskan (Thermo Scientific) plate reader. Corrected absorbance was transformed to percentage of cell viability using the following formula: *cell viability % = (Abs sample/Abs control)x100*, where *Abs* is the corrected absorbance at 570 nm after subtracting the absorbance at 590 nm.

Sample preparation for histology assays

Animals were euthanised 1 day or 30 days post injection of Ag_2S superdots or PBS (in case of controls). Organs (liver, kidneys and spleen) were extracted and placed in paraformaldehyde for tissue fixation for 72 hours. For histological studies, the tissues were embedded in paraffin blocks. In each case, 5 µm tissue sections were devoted for hematoxylin-eosin staining method.

Supplementary Note 1. Multiphoton character of the Ag_2S dot-to-superdot transformation

To investigate the multiphoton origin of the dot-to-superdot transformation, we have studied the dependence of the dot-to-superdot transformation speed on the laser pulse energy. To do so, we measured the time evolution of NIR-II fluorescence during irradiation with different laser pulse energies. For each pulse energy, the time evolution of the NIR-II luminescence was measured in its linear regime that, according to what is explained in the main text, corresponds to an initial stage of dot-to-superdot transformation in absence of laser-induced damage (**Supplementary Figure 9a**). The slope of each dI/dt curve was determined and plotted versus pulse energy in a log-log plot (**Supplementary Figure 9b**). The experimental data included in this last graph can be nicely fitted to a linear function in the log-log plot with a slope close to 2. This means that the slope dI/dt follows a power law behaviour with an exponent equal to 2, i.e. $\frac{dI}{dt} \propto E_p^2$.

We can theoretically relate the time evolution of the fluorescence intensity and the rate of dot-to-superdot transformation by writing the fluorescence intensity as:

$$I = k_{sd}\, N_{sd} + k_d\, N_d \quad \textbf{(Supplementary Equation 1)}$$

where N_{sd} and N_d is the population of superdots (dots) in the solution, and k_{sd} and k_d is a constant that depends on different parameters including the fluorescence quantum yield and the molar extinction coefficient of the superdots (dots), and on the optical collecting efficiency of the optical system used for luminescence detection. Then, time derivation of expression **Supplementary Equation 1** leads to:

$$\frac{dI}{dt} = k_{sd}\,\frac{dN_{sd}}{dt} + k_d\,\frac{dN_d}{dt} \quad \textbf{(Supplementary Equation 2)}$$

As $dN_d/dt = -\,dN_{sd}/dt$, i.e., the rate of dot annihilation is the same as the rate of super-dot creation, then:

$$\frac{dI}{dt} = (k_{sd} - k_d)\frac{dN_{sd}}{dt} \quad \textbf{(Supplementary Equation 3)}$$

Thus, for initial instants of the ultrafast laser-induced dot-to-superdot transformation, the rate at which the fluorescence intensity increases with time (dI/dt) can be considered proportional to the number of dot-to-superdot transformations per unit time, i.e. to $\frac{dN_{sd}}{dt}$. At the same time, the dot-to-superdot transformation per unit time depends on the pulse energy (E_p). As we found from the experimental data dI/dt follows a power law behaviour with an exponent equal to 2, so

$$\frac{dI}{dt} \propto \frac{dN_{sd}}{dt} \propto E_p^2 \quad \textbf{(Supplementary Equation 4)}$$

Therefore, we conclude that the dot-to-superdot transformation is triggered by a two-photon process.

Supplementary Note 2. Thermal quenching during ultrafast laser irradiation.

The ultrafast laser irradiation also leads to laser-induced heating of dispersions containing Ag_2S dots and superdots. To quantify this thermal loading, a thermocouple was placed inside the cuvette containing the irradiated dispersion. **Supplementary Figure 11a** shows the time evolution of the temperature of the dispersion containing either Ag_2S or and superdots under irradiation at 15 W cm^{-2} with 50 fs pulses. The steady state thermal loading in both cases was close to 10 ºC. The initial difference can be explained considering the non-radiative deexcitation pathways present in the Ag_2S dots that are undergoing the dot-to-superdot transformation. After a 60-minute-long irradiation, both samples presented a similar NIR-II emission brightness, as demonstrated by the fluorescence images included as an inset in **Supplementary Figure 11a**. The slightly lower intensity observed for the Ag_2S superdots can be explained considering that a second treatment on an already irradiated dispersion only promotes laser-induced thermal loading and sample damage.

We also studied the time evolution of the emitted intensity of both samples studied above (as-prepared Ag_2S dots and Ag_2S superdots) under ultrafast laser irradiation**.** As occurred for the thermal loading, the luminescence intensity of the as-prepared dispersion reached its maximum after 10 minutes and stabilized afterwards (see **Supplementary Figure 11b)**. This backs the hypothesis that the complete dot-to-superdot transformation occurs in the first 10 min of ultrafast laser irradiation.

The decrease in the fluorescence signal generated by already transformed Ag_2S superdots can be attributed to the thermal quenching caused by the laser-induced thermal loading. In order to corroborate this hypothesis, we measured the temperature dependence of Ag_2S superdots (**Supplementary Figures 11c-d**). The experimental data revealed a temperature-induced intensity quenching close to 4% °C^{-1}. Considering the maximum temperature increment of 10 ºC observed for the superdot dispersion, we can estimate that the thermal quenching causes a decrease in the emission intensity of around 40%. This is quite close to the intensity reduction obtained during irradiation of Ag_2S superdots (≈ 50%, as obtained from experimental data included in **Supplementary Figure 11b)**. This fact suggests that the observed intensity reduction is mainly caused by the interplay between laser-induced thermal loading and the intrinsic thermal quenching of the NIR-II emission of Ag_2S.

Supplementary Note 3. Thermal loading of dots and superdots.

Supplementary Figures 13a-c show the optical, NIR-II and thermal images of dispersions of Ag_2S dots in $CHCl_3$ subjected to ultrafast laser illumination processes of durations varying from 0 to 90 min. Both fluorescence and thermal images were obtained under 808 nm continuous wave (CW) illumination with a power density of 0.3 W cm^{-2}. **Supplementary Figure 13d** shows the evolution of both the NIR-II emission intensity and the laser-induced temperature increment as a function of the irradiation time. The NIR-II fluorescence intensity increases with the irradiation time, in agreement with the increment in the QY detailed in the main text. At the same time, the laser-induced temperature increment decreases with the irradiation time. Ag_2S superdots show a laser-induced thermal loading close to 50% of that observed for Ag_2S dots. This could be caused by a lower absorbance at 808 nm and/or by the reduced probability of non-radiative deexcitation. From **Supplementary Figure 13d**, we conclude that the slight (20%) reduction in the absorbance at 808 nm triggered by the dot-to-superdot transformation cannot explain the remarkable reduction in the thermal loading. This suggests that such reduction is caused by the increase in the fluorescence QY. The temperature increment can be considered, as a first order approximation, to be proportional to the fractional thermal loading, η_T. This is defined as the fraction of the absorbed pump power that is transformed into heat *via* nonradiative decay. The QY and η_T are related through the following expression:

$$\eta_T = 1 - QY * \left(\frac{\lambda_{exc}}{\langle\lambda_{em}\rangle}\right) \qquad \text{(Supplementary Equation 5)}$$

where λ_{exc} is the excitation wavelength (808 nm in our case) and $\langle\lambda_{em}\rangle$ is the average emission wavelength (1220 nm in our case). Thus, the large increment in the QY due to ultrafast laser irradiation is here identified as the main cause of the remarkable reduction in the laser-induced thermal loading.

Supplementary Note 4. Influence of skin thickness variations on *in vivo* image contrast.

The depth of a subcutaneous injection is not fully controllable even when following identical protocols. In our experiments, special care was taken to ensure that the dispersions containing the NIR-II contrast agents were injected between the inner face of the skin (dermis + epidermis + hypodermis) and the muscular wall.

In these conditions, the possible variation in the injection depth is caused by the non-homogeneous thickness of the skin. According to the literature, the average thickness of the skin of a mouse is close to 400 µm[1]. The variation in skin thickness of mice is close to 80 µm. Based on this thickness variation, we can estimate the magnitude of the fluctuation in the detected fluorescence intensity. The subcutaneously injected NIR-II probes generate a fluorescence intensity I_0. According to the Lambert-Beer law, the fluorescence intensity that is transmitted through the skin and, therefore, can be detected by the fluorescence camera, I_{det}, is given by:

$$I_{det} = I_0 exp\left[-d \cdot \alpha_{ext,skin}\right] \quad \textbf{(Supplementary Eq. 6)}$$

where $\alpha_{ext,skin}$ is the optical extinction coefficient of skin that, in the NIR-II, is close to 25 cm^{-1} . In Supplementary Eq. 6, d = 400 µm is the skin thickness. Then, a change in the skin thickness of Δd = 80 µm will cause a change in the detected intensity, ΔI_{det}, that can be estimated by:

$$\Delta I_{det} = I_0 exp\left[-d \cdot \alpha_{ext,skin}\right] \frac{d}{\alpha_{ext,skin}} = -I_{det}\left[\Delta d \cdot \alpha_{ext,skin}\right] = -0.2 \cdot I_{det} \quad \textbf{(Supplementary Eq. 7)}$$

This indicates that the uncertainty in the injection depth due to variations in skin thickness is close to 20%, which is well below the observed differences in fluorescence intensities.

When comparing the *in vivo* images obtained after subcutaneous injection of the different NIR-II probes, we observe that the contrast of the fluorescence images obtained with superdots is several orders of magnitude above those obtained for any other NIR-II fluorescent probe. Therefore, the possible variations in injection depth cannot explain the much better performance of superdots, which is then attributable to their superior brightness.

Supplementary Note 5. Tissue penetration depth of Ag_2S fluorescence

To determine the penetration depth of Ag_2S superdots in our NIR-II imaging setup, we performed a simple experiment placing a piece of biological tissue (chicken breast) on top of a quartz cuvette filled with a dispersion of Ag_2S superdots. The tissue was cut to resemble a right triangle (**Supplementary Fig. 17a**). When illuminating the whole system, a single fluorescence image (see **Supplementary Fig. 17b**) allows obtaining the values of luminescence intensity at different depths (**Supplementary Fig. 17c**). With this set of data, it is possible to estimate the traditional penetration depth, l_{trad}, and the extrapolated penetration depth, l_{max}, of the luminescence. To do this, the experimental data are fitted to $I(l) = I_0 + \Delta I \exp(-l/l_{trad})$, where l is the tissue thickness, I_0 is the background intensity detected by the NIR-II camera when the sample is not being excited and ΔI is the fluorescence signal increment caused by the excited cuvette with no tissue on top. The traditional penetration depth l_{trad} is then directly obtained with the fitting and corresponds to the tissue thickness that decreases the luminescence intensity down to $I_0 + {}^{\Delta I}/_{e}$.The extrapolated penetration depth, however, is found by the expression $l_{max} = l_{trad}\, Ln({}^{\Delta I}/_{\sigma_{noise}})$, where σ_{noise} is the intrinsic noise of the measurement. l_{max} , therefore, corresponds to the highest depth at which a signal greater than the intrinsic noise of the measurement can be detected.

The dependence of l_{trad} and l_{max} on laser power density is shown in **Supplementary Fig. 17d**. While l_{trad} presents a constant value of 3.2 mm, l_{max} follows a logarithmic trend, increasing from 1 to 2 cm. These results indicate that Ag_2S superdots allow for fluorescence imaging into tissues at depths larger than 1 cm under our experimental conditions.

Supplementary Note 6. Effect of camera performance on image quality.

Supplementary Fig. 18 shows some previously reported NIR-II *in vivo* fluorescence images of mouse vasculature obtained with different NIR-II probes and detection systems that are summarized in **Supplementary Table 3**. Some of these images outperform ours in terms of image resolution and/or contrast. To explain these differences, we have analysed the experimental conditions reported in each case. The majority of the fluorescence images shown in Supplementary Fig. 18 were obtained with high-end NIR-II cameras cooled down to at least -80 °C (see **Supplementary Table 3**). This deep cooling reduces the dark noise of the camera, as indicated in Supplementary Table 3. The NIR-II used in our work has a slightly higher noise due to operating at a higher temperature (-40 ºC). There is also a difference in pixel size, which is larger in our case (30x30 μm^2) than in higher-end cameras (typically, 20x20 μm^2). This has a direct impact on the spatial resolution. Thus, differences in camera performance could explain the observed differences in quality between our images and those reported by other groups using Ag_2S dots.

Supplementary Note 7. Subchronic toxicology assays

For at least 28 days after administration, all hepatic enzymes levels fall into the normal range for mice as provided by the reference laboratory (see **Supplementary Fig. 20**)[2]. These ranges are relative broad as they are obtained from mice of different strains, age and gender, all of which affects the expression levels of these enzymes to some extent. The fact that the enzyme levels obtained were within these normal ranges does not ensure the absence of liver damage. Nevertheless, the ALT levels remain stable for the duration of the experiment, which is especially relevant as even though ALT, ALP and AST are biomarkers of liver damage, only ALT is found primarily in the liver. ALP is found in the liver and bones whereas AST is found in the liver, kidneys, brain, heart and skeletal muscle. Therefore, it is accepted that preliminary detection of hepatocellular injuries can be elucidated by just monitoring alterations in the ALT enzyme[3]. Therefore, stable ALT levels are indicative of absence of relevant hepatic damage. While we observed changes in the ALP and AST levels, these still fall within normal ranges. The observed alterations in these enzymes can be tentatively explained considering the stress induced by the manipulation of the animals during the treatment, which reportedly affects the hepatic metabolism and thus its enzymatic levels.

Further, no creatinine or bilirubin elevations were observed (see **Supplementary Fig. 21).** This indicates the kidney function is not affected and that there is no liver damage or significant destruction of erythrocytes, as bilirubin is a hemoglobin metabolite excreted *via* the bile. All these results are consistent with the histopathological studies (see Supplementary Fig. 23) and suggest that, despite preferential accumulation in the liver, Ag_2S superdots do not exert a significant cytotoxic effect for at least 28 days after treatment. These data are in accordance to that reported elsewhere for Balb/c mice injected with 15 mg kg^{-1} dose of Ag_2S dots[11]. However, further experiments would be required to consider Ag_2S superdots fully biocompatible.

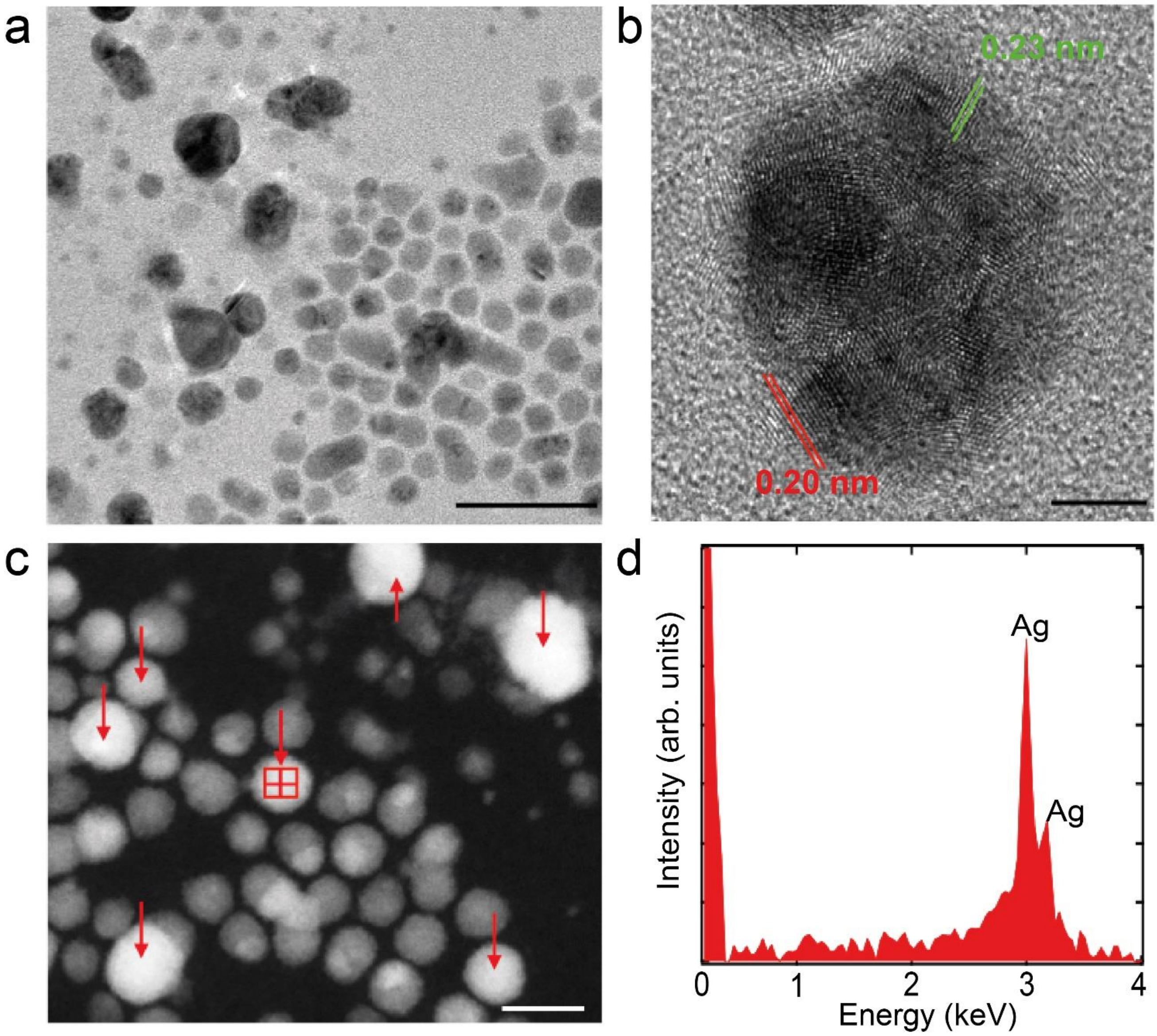

Supplementary Figure 1. Side-products of ultrafast laser irradiation of Ag_2S dots. **a** TEM image of the as-synthesized Ag_2S dots, showing the coexistence of two types of NPs with different electron-density. Scale bar is 50 nm. **b** HR-TEM of a single electron-dense NP, showing its polycrystalline structure with lattice fringes 0.20 and 0.23 nm that correspond to the 200 and 111 planes of cubic Ag. Scale bar is 5 nm. **c** HAADF-STEM image of the as synthesized Ag_2S dots highlighting the difference in Z-contrast between Ag NPs (labelled with red arrows) and Ag_2S dots. Scale bar is 20 nm. **d** EDS elemental analysis obtained from the high Z-contrast NP labelled with the red crosshair.

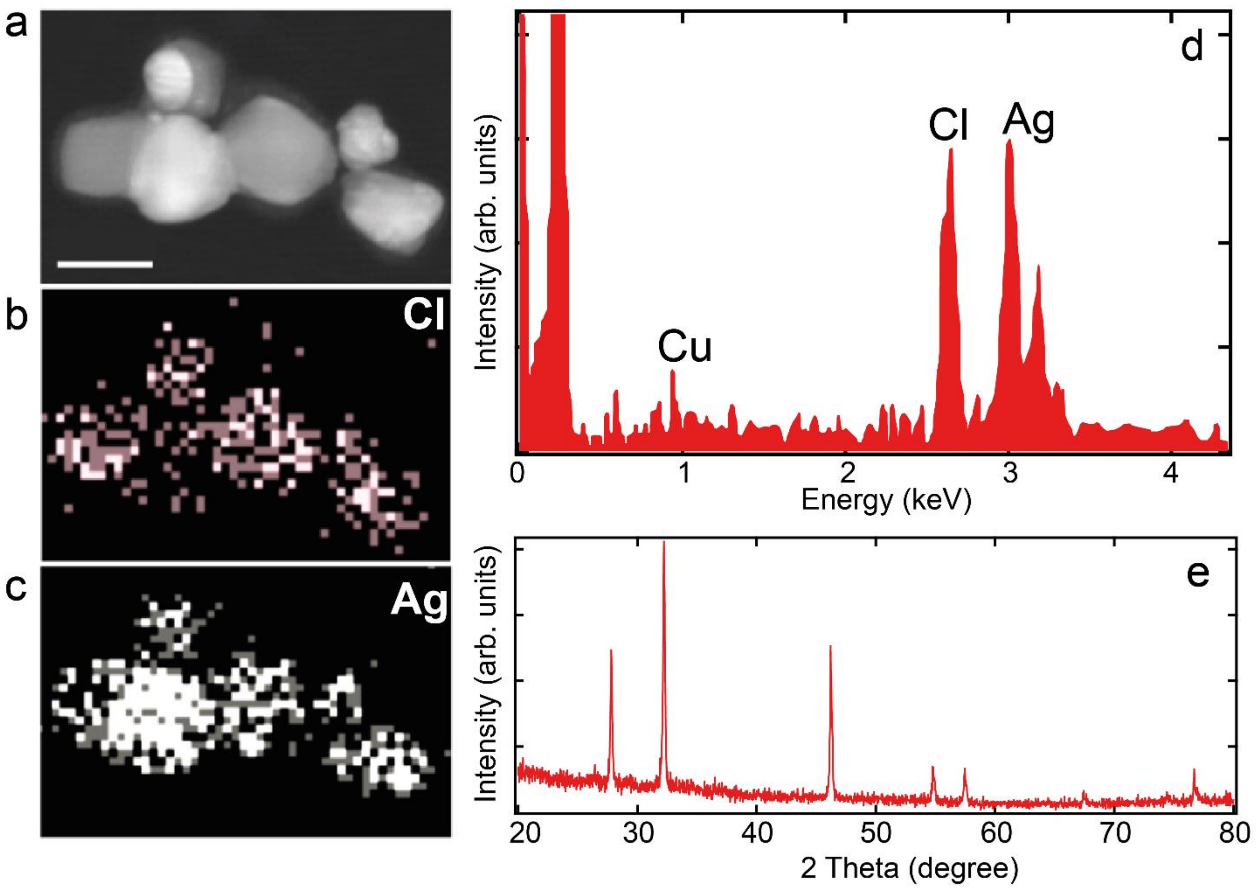

Supplementary Figure 2. Characterization of AgCl nanoparticles. a STEM micrograph of AgCl NPs obtained by ultra-fast laser irradiation of the sample. Scale bar is 50 nm. 2D EDS mapping of the spatial distribution of Cl (**b**) and Ag (**c**) reveals that these NPs are basically constituted by AgCl. This result is confirmed by EDS analysis in **d**. **e** XRD analysis of the sample shows the typical reflection of cubic AgCl crystals. After ultrafast laser irradiation, the Ag NPs in the sample react with the solvent ($CHCl_3$) generating a side product, which appears as a white powder easily removable by centrifugation at low speeds (5000 rpm).

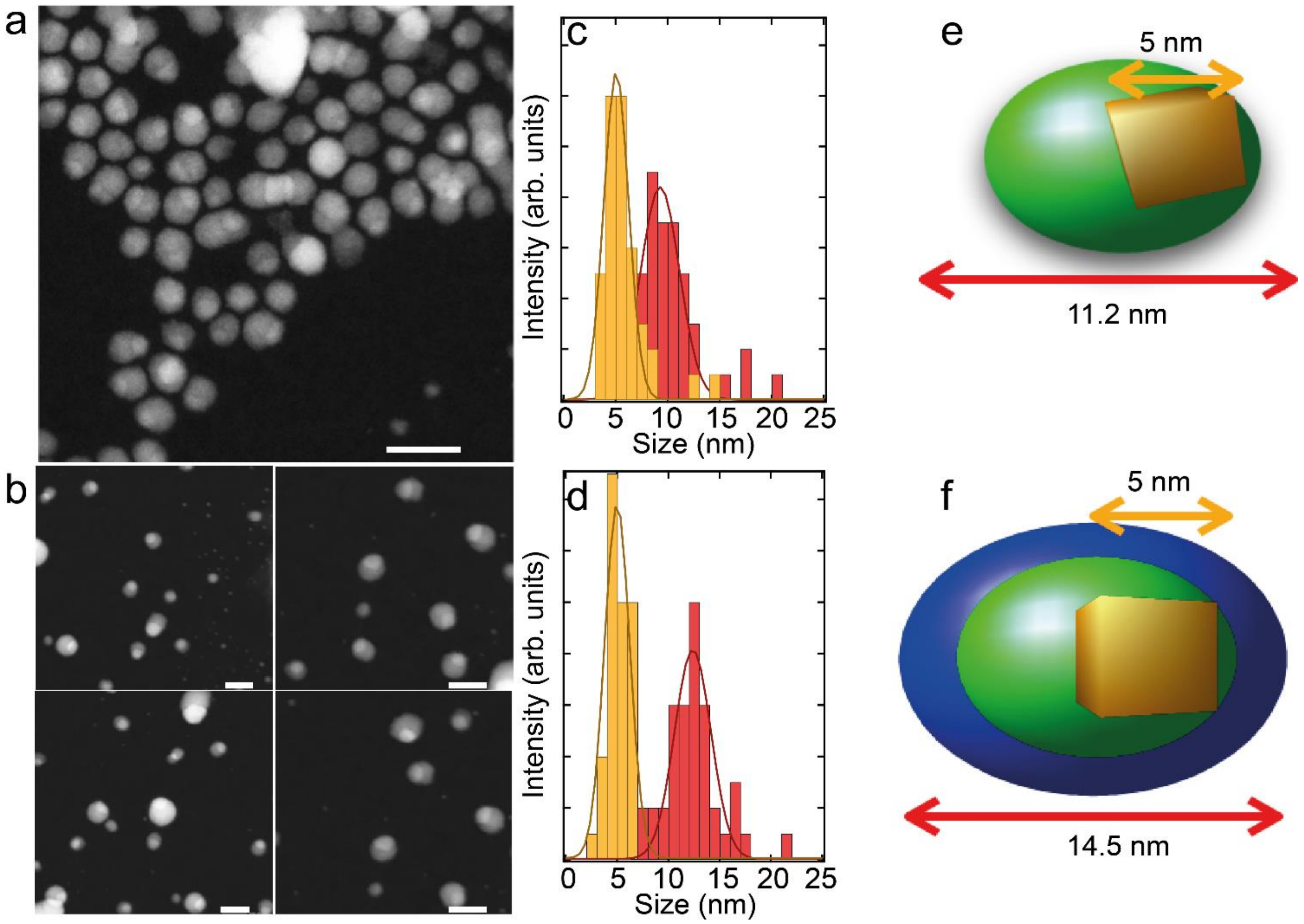

Supplementary Figure 3. Morphology of Ag_2S dots before and after ultrafast laser irradiation. HAADF-STEM image of Ag_2S dots (**a**) and superdots (**b**). Scale bars are 20 nm. Size distributions of Ag_2S dots (**c**) and superdots (**d**). Orange and red bars correspond to the size distribution of the Ag core and of the NP, respectively. **e** Schematic representation of the structure of Ag_2S dots. The average sizes of the NP and the Ag core are indicated. **f** Schematic representation of the structure of Ag_2S superdots, including the sizes.

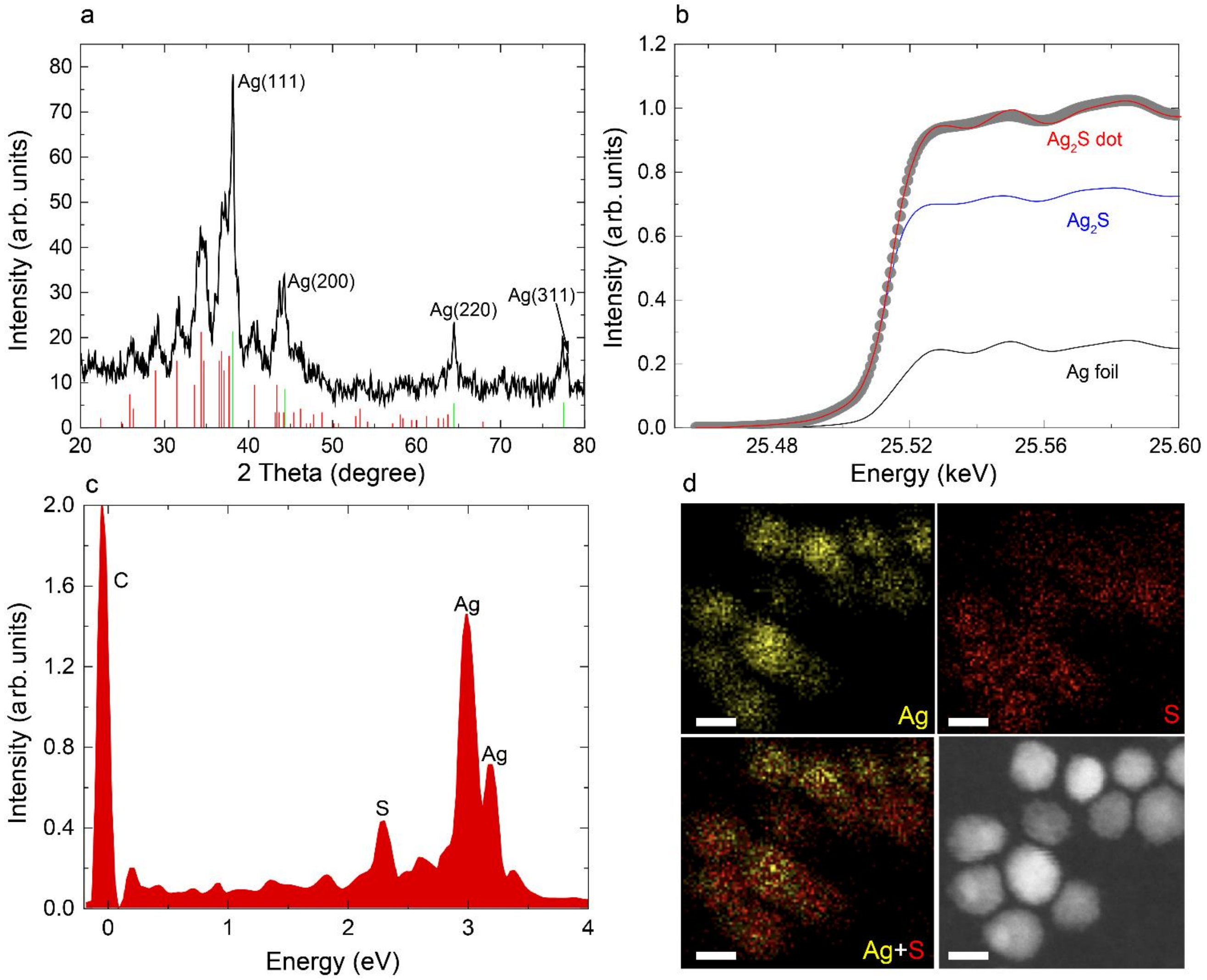

Supplementary Figure 4. Chemical and structural characterization of Ag_2S dots. a XRD pattern of as-synthesized Ag_2S dots. The red lines represent typical reflections at 34.385° (−121) and 36.556° (112) of monoclinic Ag_2S phase (JCPDS card No. 14-0072; lattice constants: a = 4.229 Å, b = 6.931 Å, c = 7.862 Å). The green lines represent the reflection positions of cubic Ag (111), (200), (220) and (311) (JCPDS card No. 04-0783; lattice constants: a = 4.0862 Å). **b** XANES spectra of Ag_2S dots (red) and Ag_2S used as reference (blue) and Ag foil used as reference (black), indicating that 25% of the total Ag content is metallic Ag, while the remainder (75%) is Ag^+ in Ag_2S. **c** EDS spectrum of Ag_2S dots. The atomic percentage of S and Ag (both Ag^0 and Ag^+) in the sample are 26% and 74%, respectively. Considering the Ag_2S stoichiometry, each S atom should be bound to 2 Ag^+ ions. Thus, from the total content of Ag (74%), 52% would correspond to Ag^+ ions forming Ag_2S and the remaining 22% would correspond to metallic Ag. **d** EDS elemental mapping of Ag_2S: silver (top left, yellow), sulphur (top right, red), merge of silver and sulphur (bottom left). HAADF-STEM image from which the EDS map was collected (bottom right). The EDS map shows silver-enriched regions that match the areas of high Z-contrast in the HAADF-STEM image. Scale bars are 10 nm.

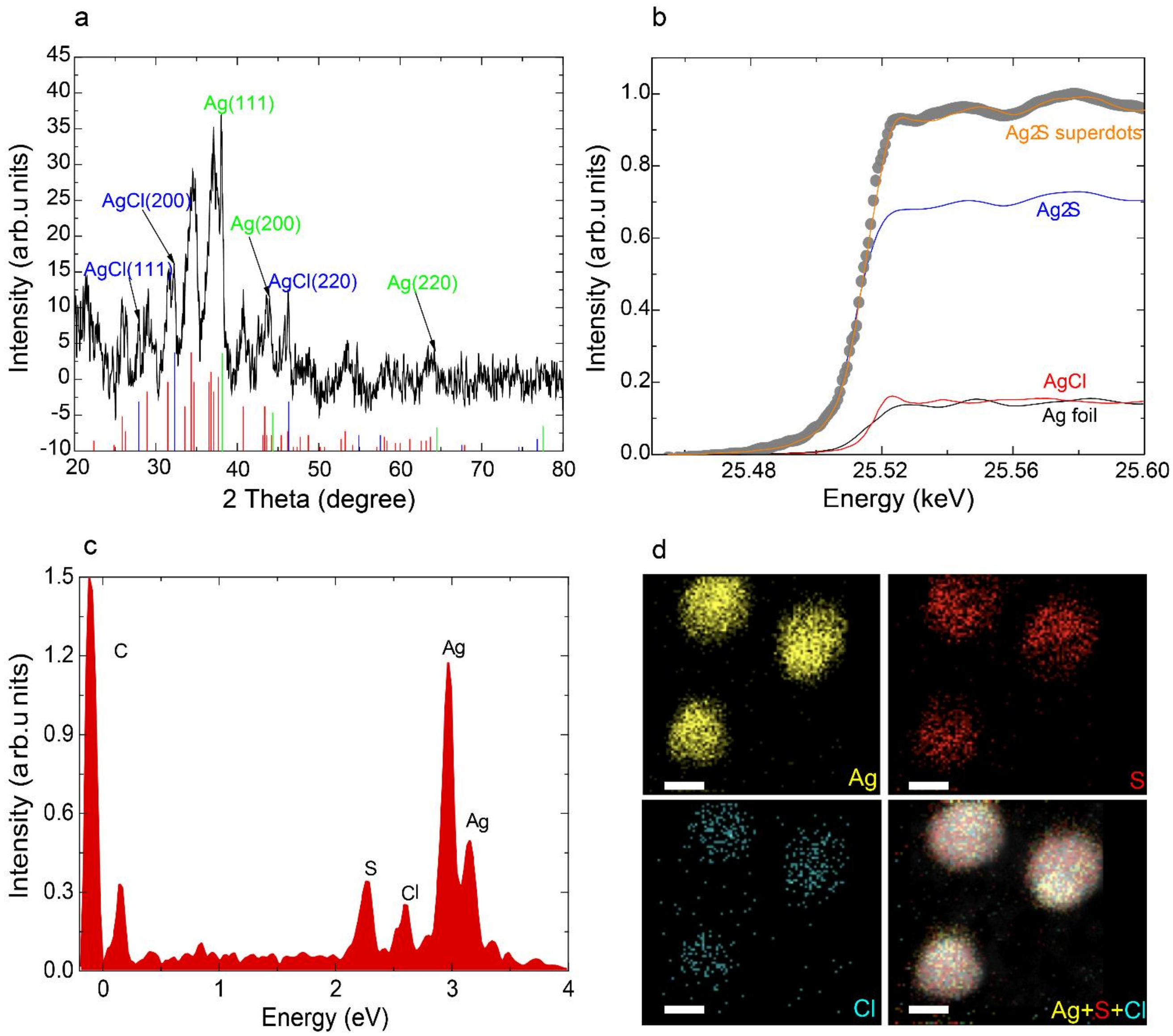

Supplementary Figure 5. Chemical and structural characterization of Ag_2S superdots. a XRD pattern of Ag_2S superdots, showing peaks corresponding to monoclinic Ag_2S (JCPDS card No. 14-0072; lattice constants: a = 4.229 Å, b = 6.931 Å, c = 7.862 Å) and cubic Ag (JCPDS card No. 04-0783; lattice constants: a = 4.0862 Å) as labelled. Tiny reflections at 32.312° (200), 46.138° (220) and 55.262° (311) can be attributed to cubic AgCl (JCPDS file 31-1238; lattice constant a= 5.549 Å). **b** XANES spectra of Ag_2S superdots (orange) and Ag_2S used as reference (blue), AgCl used as reference (red) and Ag foil used as reference (black). The percentage of Ag^+ stemming from Ag_2S phase remained around 70% after ultrafast laser illumination, while that of Ag^0 was reduced from 22% to 14%. A component of Ag^+ as AgCl is present in a percentage close to 16%, indicating the transformation of Ag^0 towards Ag^+. **c** EDS spectrum of Ag_2S superdots. The overall atomic composition of the sample is 67% silver, 12% sulphur and 12% chlorine. Considering the Ag_2S stoichiometry, the Ag forming Ag_2S phase would be 42% out of the total 67%. 12% of Ag atoms would form AgCl (since the atomic percentage of Cl is 12%) and the remaining 13% of Ag atoms would correspond to Ag^0. **d** EDS elemental mapping of Ag_2S superdots, silver (yellow), sulphur (red) and chlorine (blue). The inset in the bottom right is the resulting image after merging the S, Ag and Cl HAADF-STEM images. The Cl atoms are homogeneously distributed throughout the whole nanostructure, which would indicate the presence of AgCl phase in the whole NP. Scale bars are 10 nm.

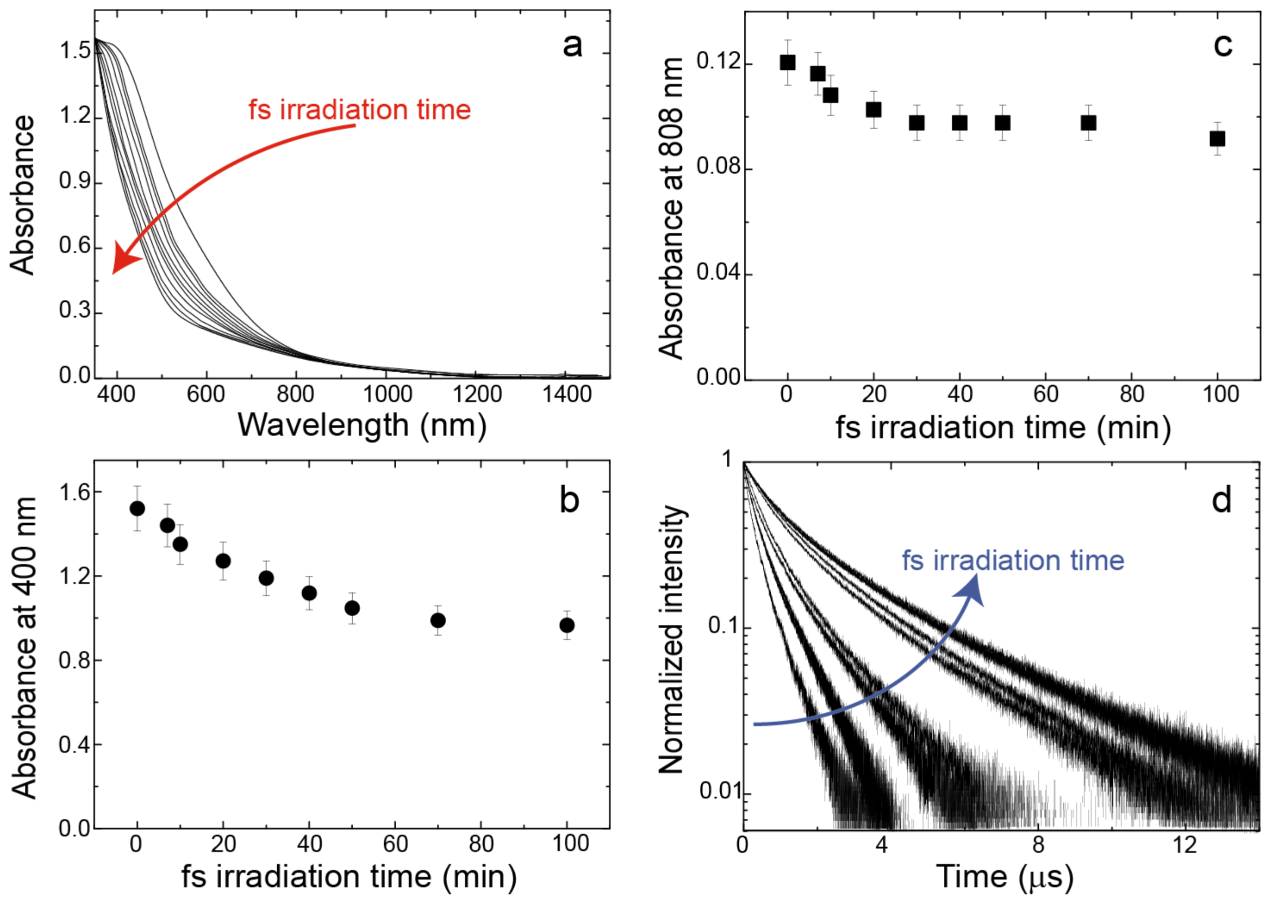

Supplementary Figure 6. **Effect of the duration of the ultrafast laser irradiation on the properties of Ag_2S dots. a** VIS-NIR extinction spectra of colloidal dispersions of Ag_2S dots in $CHCl_3$ after ultrafast laser irradiation for different times. **b** Extinction coefficient at 400 nm as a function of the ultrafast laser irradiation time, obtained from the spectra in **a**. **c** Extinction coefficient at 808 nm as a function of the ultrafast laser irradiation time, as obtained from **a**. **d** Fluorescence decay curves of colloidal dispersions of Ag_2S dots in $CHCl_3$ after ultrafast laser irradiation for different times. The irradiation pulse width and power density (50 fs, 9 W cm^{-2}) were identical in all cases.

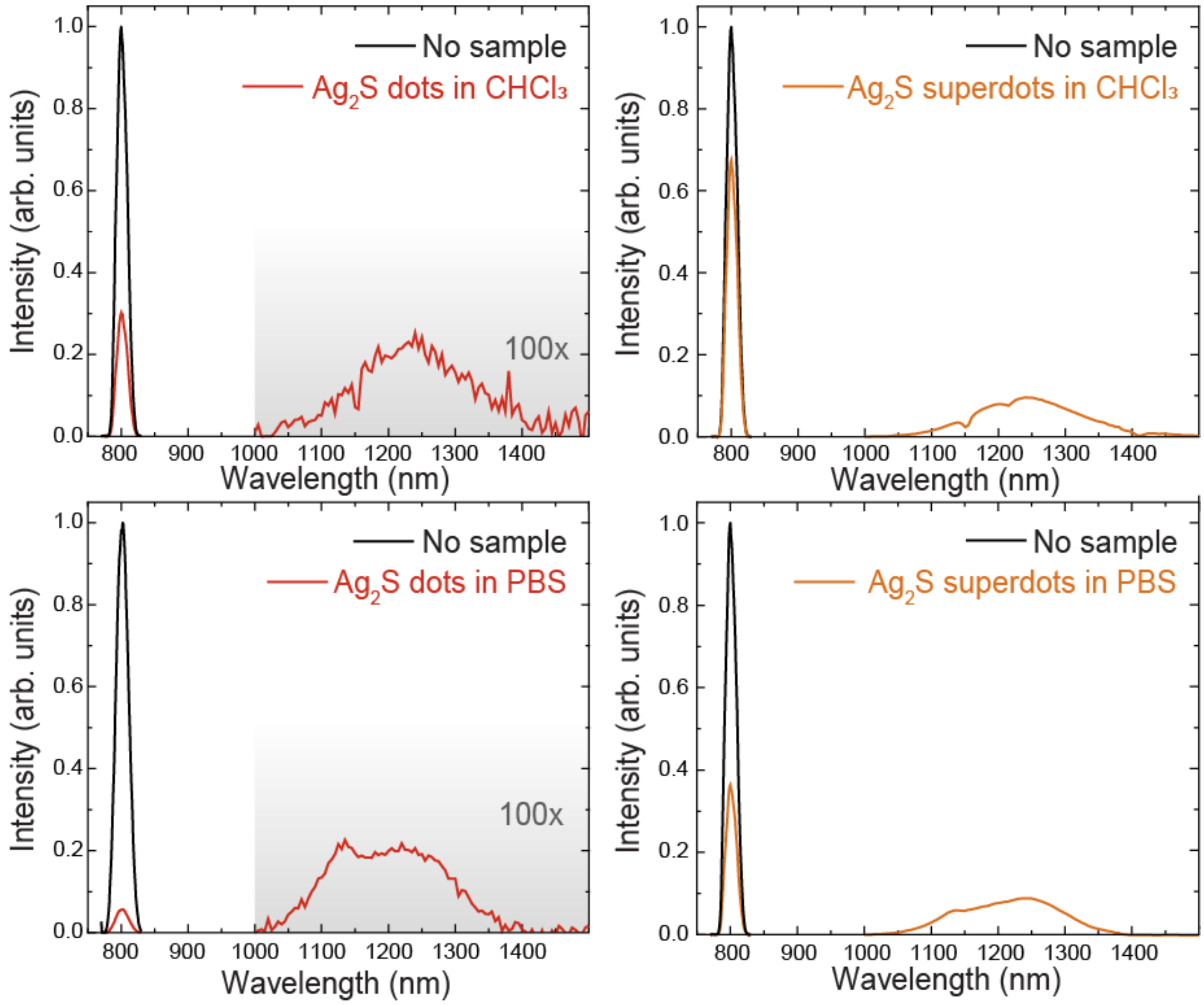

Supplementary Figure 7. Experimental data for quantum yield calculations. Illustrative examples of the spectra used for the determination of the QY. Data correspond to dispersions of Ag_2S dots and superdots in $CHCl_3$ and in PBS. The spectrum of the excitation light (808 nm) is measured with and without sample. From the difference between these spectra, the number of absorbed photons is calculated in each case. The number of emitted photons generated by each sample is calculated from the emission spectra recorded in presence of the sample. Then, the QY is calculated by dividing the number of emitted photons by the number of absorbed photons. All the emission spectra are corrected by the system response.

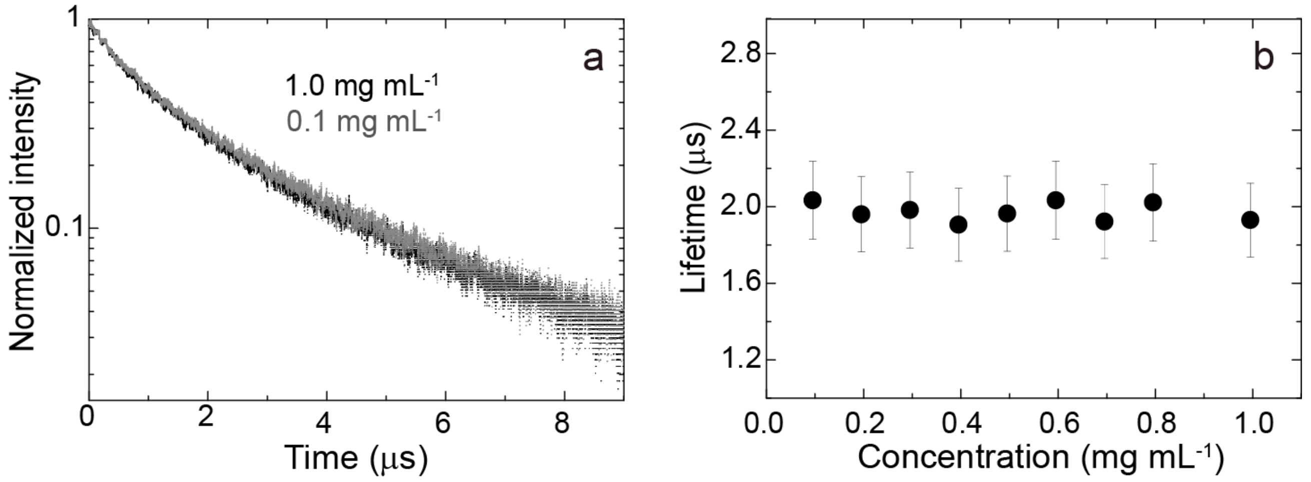

Supplementary Figure 8. **Concentration dependence of emission lifetime. a** Fluorescence decay curves from colloidal dispersions of Ag_2S dots in $CHCl_3$ after ultrafast laser irradiation (50 fs, 0.6 W and 9 W cm^{-2}) for 90 min at concentrations of 1.0 and 0.1 mg mL^{-1}. **b** Lifetime as a function of the concentration of the laser-irradiated dispersion of Ag_2S dots. The Ag_2S superdot dispersion was diluted from 1 mg mL to 0.1 mg mL^{-1} to determine whether any artefacts related to high concentration of Ag_2S superdots in $CHCl_3$ were present. No evidence of concentration-related artefacts was found in the lifetime measurements, demonstrating that the long lifetime of Ag_2S superdots is achieved by minimizing non-radiative pathways as the ultrafast laser treatment is applied. Dots correspond to the averaged value obtained after 5 measurements in each conditions. Error bars are the standard deviation calculated in each case.

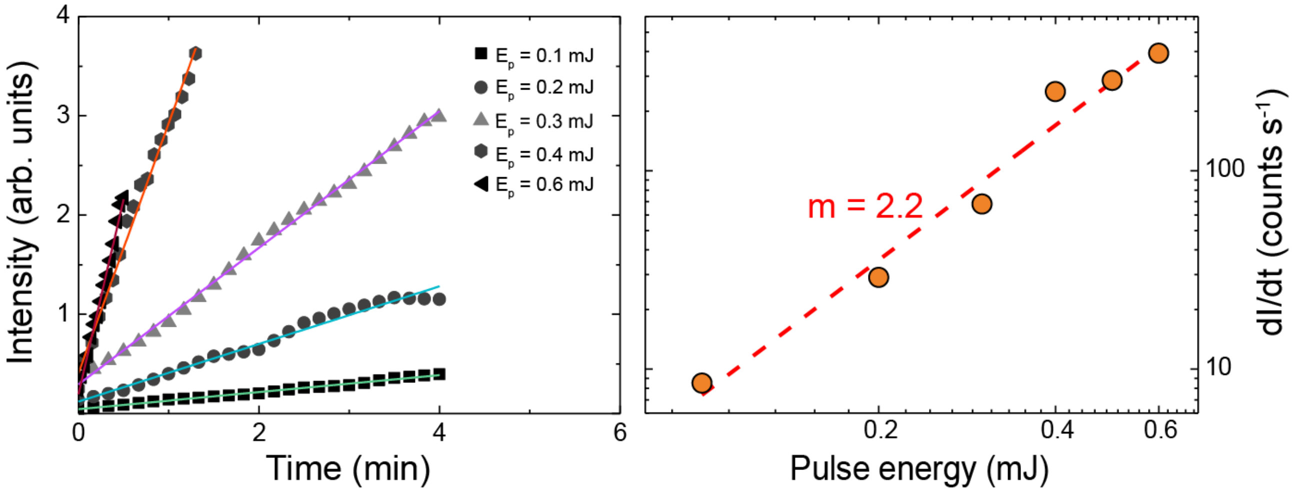

Supplementary Figure 9. Multiphoton character of the Ag_2S dot-to-superdot transformation. a Time dependence of the NIR-II fluorescence generated by a colloidal solution of Ag_2S dots in chloroform under irradiation with 50 fs, 800 nm laser pulses of different energies. **b** The time derivative of intensity as a function of irradiation pulse energy (E_p). The slope of the curve indicates that η is directly proportional to E_p^2 so that it is a two-photon process.

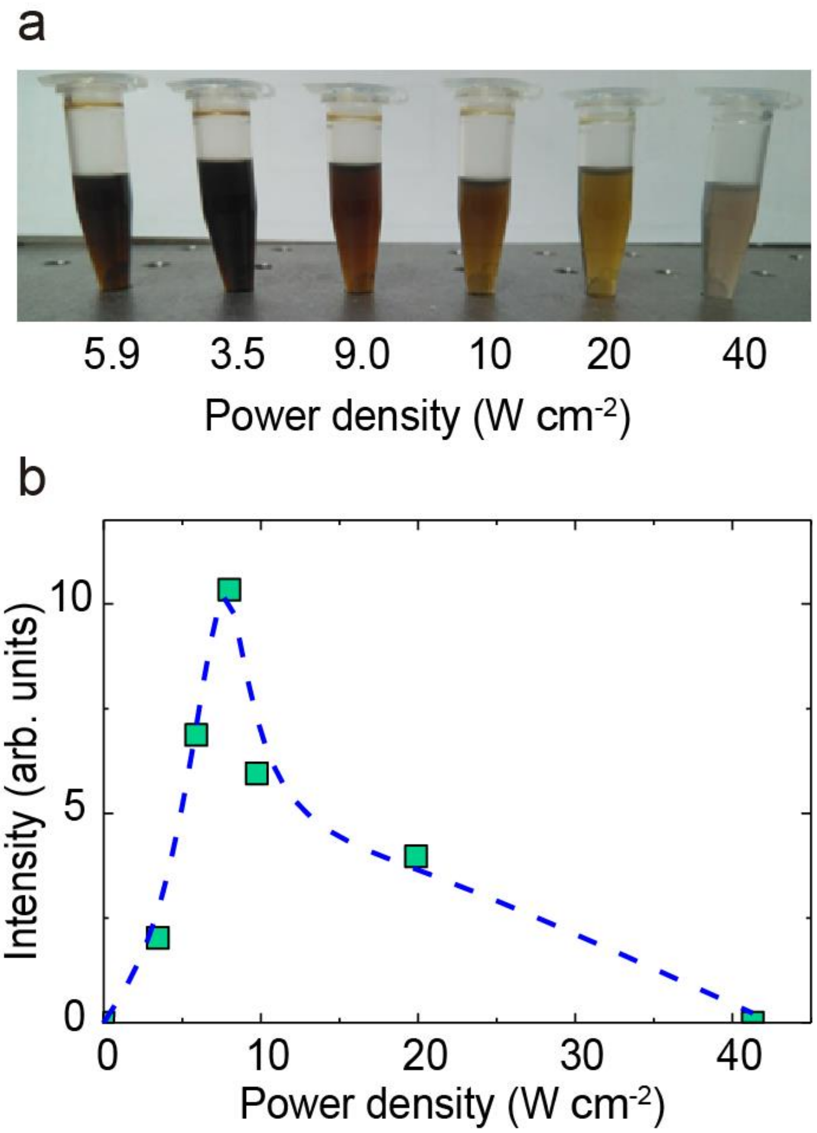

Supplementary Figure 10. **Effect on irradiation power density on the dot-to-superdot transformation.** Optical images (**a**) and NIR-II emission intensity (**b**) for a dispersion of Ag_2S in $CHCl_3$ after a 100-minute-long ultrafast laser irradiation at different power densities.

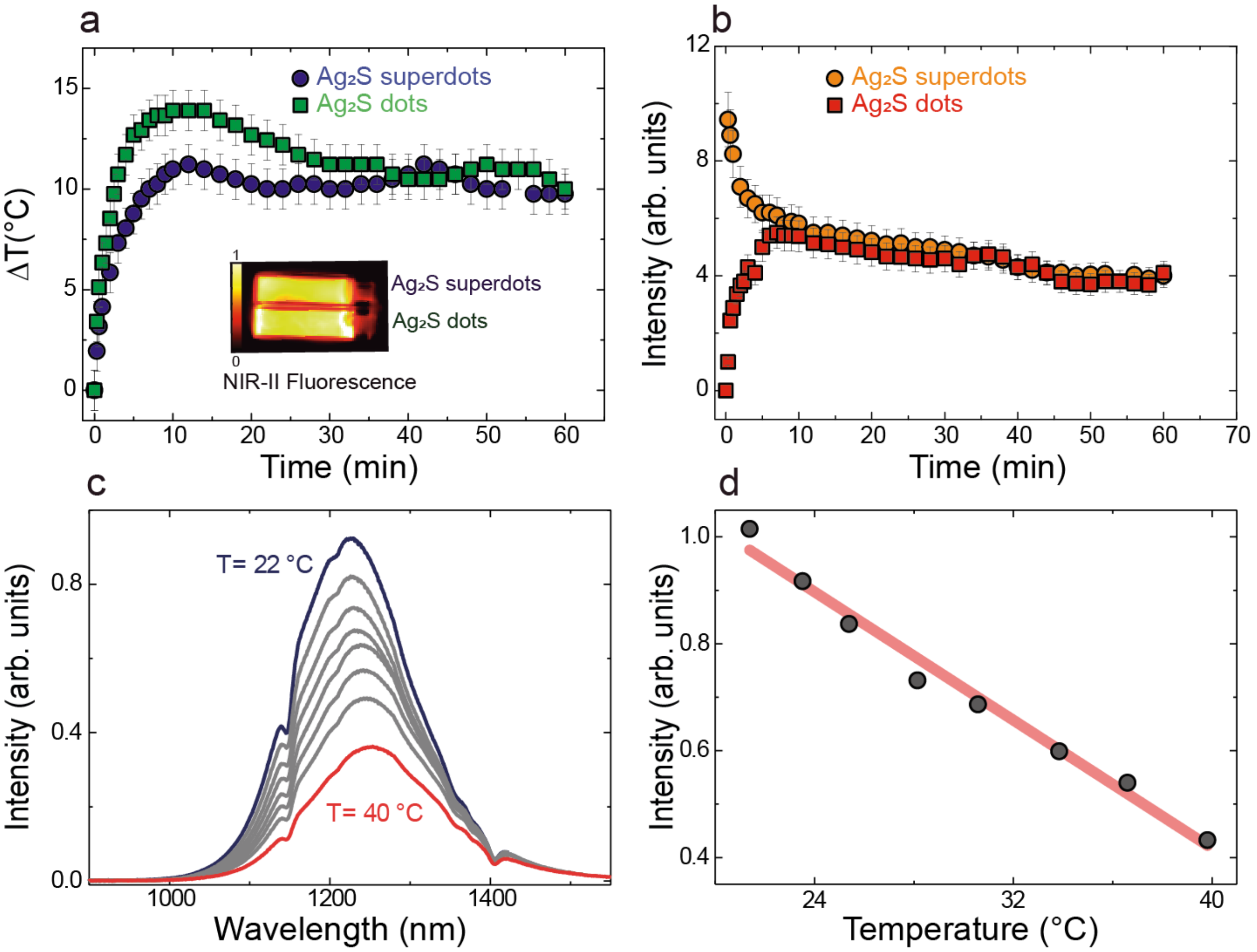

Supplementary Fig. 11. **Thermal quenching during ultrafast laser irradiation. a** Time evolution of temperature increment for Ag_2S dots and Ag_2S superdots dispersed in $CHCl_3$ during ultrafast laser irradiation with 50 fs laser pulses during 60 min at a fluence of 15 W cm^{-2}. **b** Time evolution of the emitted intensity generated from both dispersions during the same treatment applied as described in **a**. Experiments were replicated three times. Symbols correspond to the average value. Error bars in **a** and **b** are the resolution of thermal camera and the intensity fluctuations of the infrared spectrometer. **c** Temperature dependence of the emission spectrum of Ag_2S superdots, as measured under optical excitation with 808 nm light. The temperature dependence of the emitted intensity extracted from the spectra in **c** is shown in **d**.

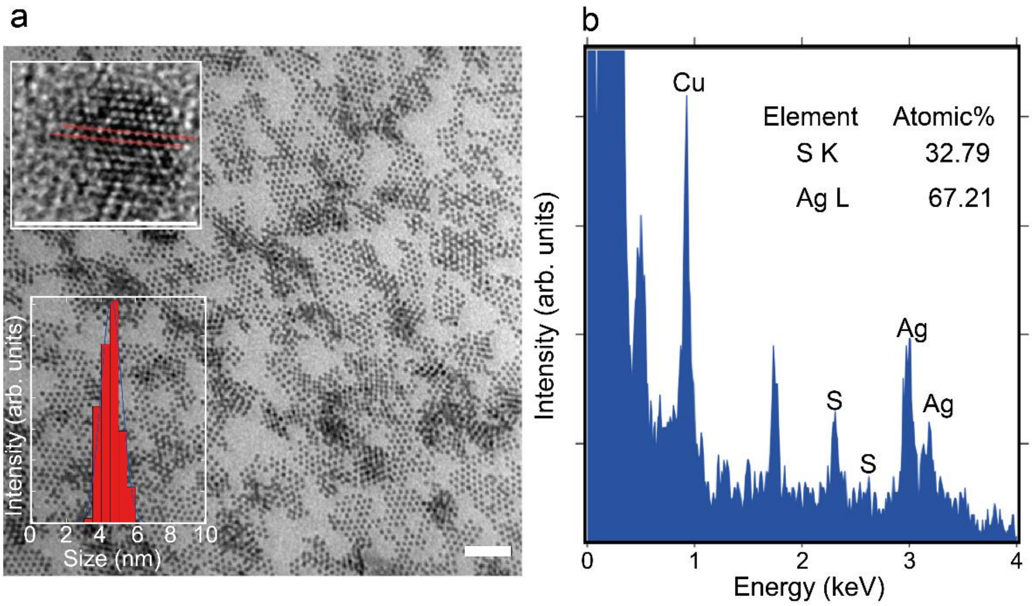

Supplementary Figure 12. Characterization of neat Ag_2S dots. a TEM micrograph of the neat Ag_2S dots produced by thermal decomposition of AgDDTC. The scale bar is 50 nm. The upper inset (scale bar: 5 nm) depicts a HR-TEM of a single NP, showing the lattice fringe d_{022}=2.5Å. The lower inset shows the size distribution of the dots, centred at 4 nm of diameter**. b** EDS spectrum of the neat Ag_2S dots, indicating a Ag:S atomic ratio of 2:1, characteristic of Ag_2S nanocrystals.

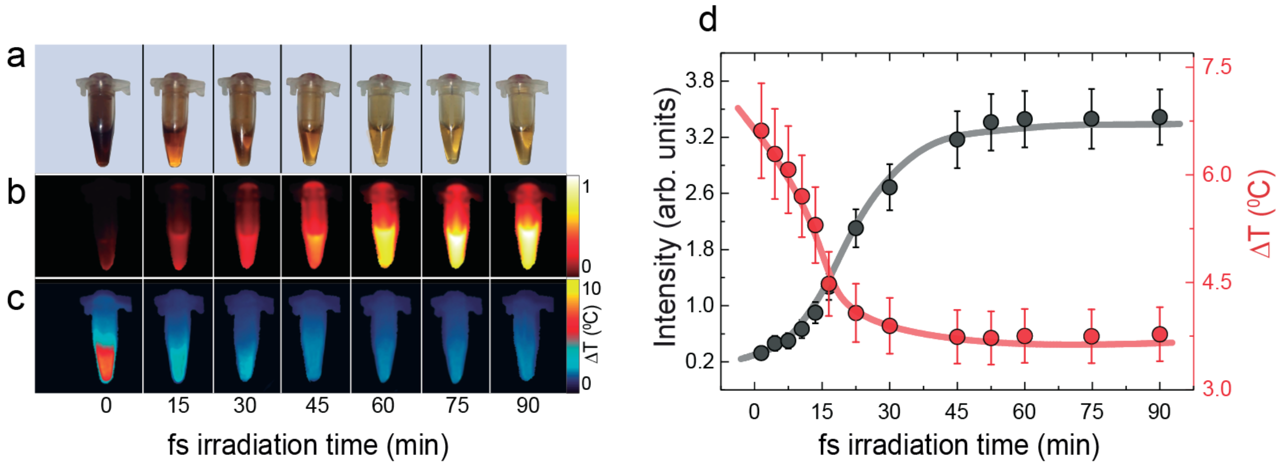

Supplementary Figure 13. **Thermal loading of Ag_2S dots and superdots.** Optical (**a**), NIR-II fluorescence (**b**) and thermal images (**c**) obtained from colloidal dispersions of Ag_2S dots in $CHCl_3$ after ultrafast laser irradiation (50 fs, 9 W cm^{-2}) for times in the 0-90 minute range. **d** NIR-II emission intensity and temperature increment for of Ag_2S dot dispersions subjected to different durations of ultrafast laser irradiation, as shown in **a**. Error bars in **d** are given by the thermal resolution of the thermal camera and by the intensity fluctuations of the infrared camera used for the acquisition of fluorescence images.

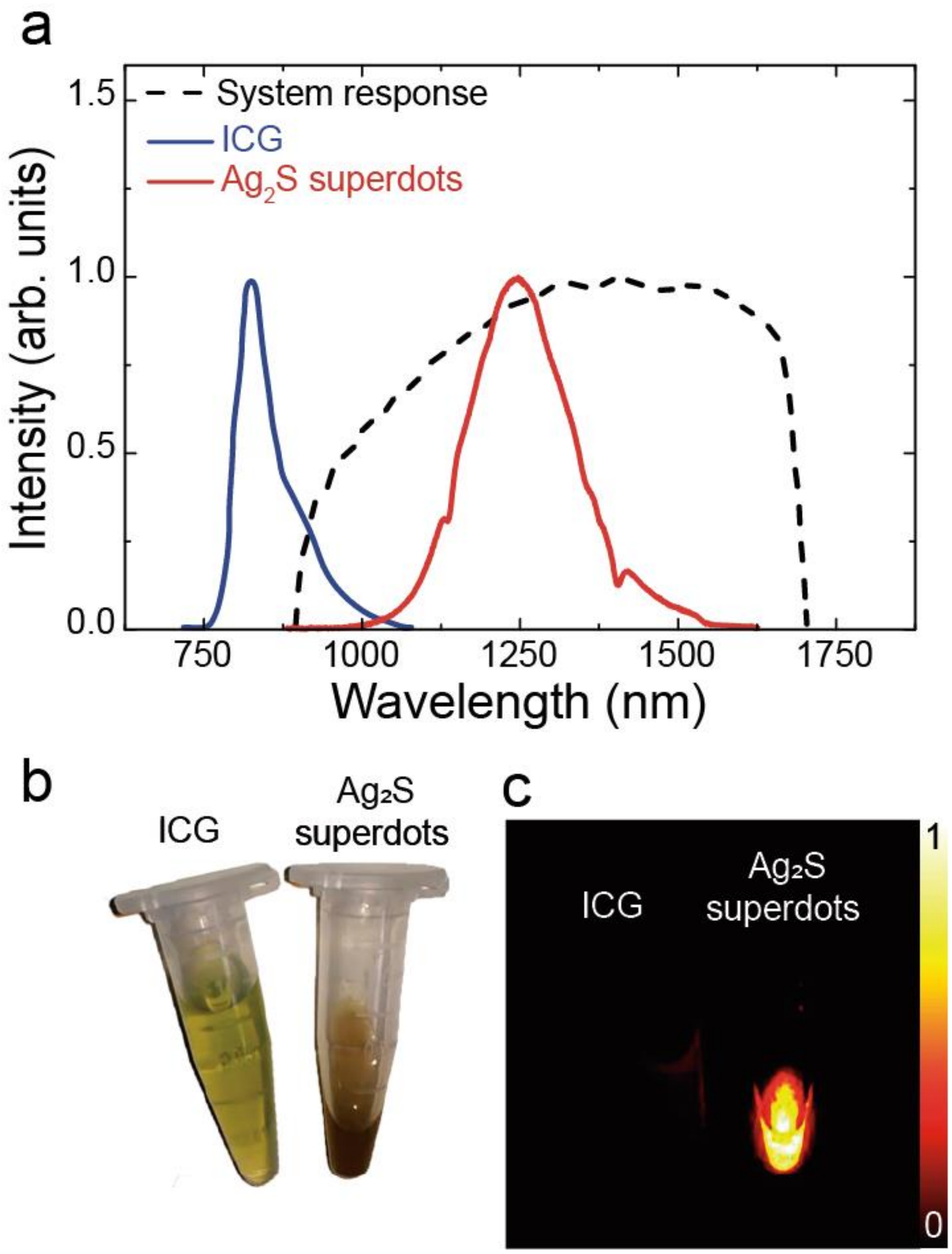

Supplementary Figure 14. **Comparison of ICG and Ag_2S superdots as NIR-II probes. a** Normalized emission spectra of ICG and Ag_2S superdots plotted together with the spectral response of our NIR-II imaging system, showing the poor overlap between the tail of the ICG emission and the spectral response of our detector. **b** Optical and NIR-II fluorescence images of colloidal dispersions of ICG and Ag_2S superdots. A brighter emission is observed for Ag_2S superdots.

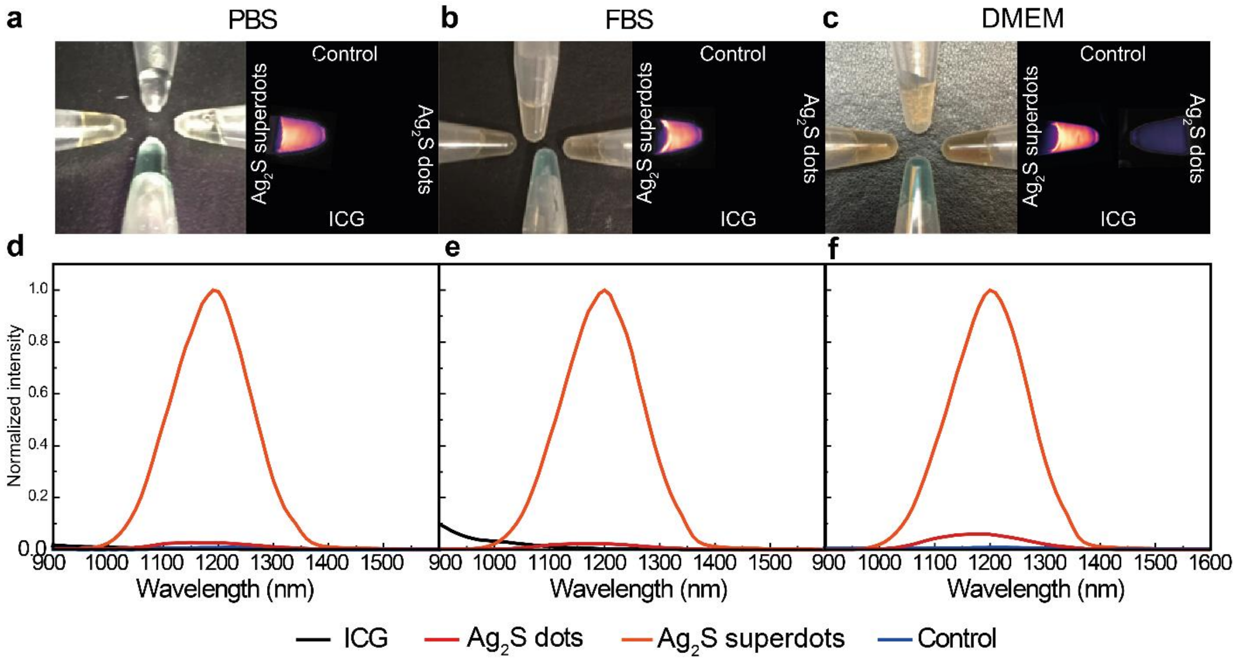

Supplementary Figure 15. Emission of Ag_2S superdots and other NIR-II probes dispersed in different media. Optical and fluorescence images of Eppendorf tubes filled with either a control solution or dispersions of ICG, Ag_2S dots or Ag_2S superdots in PBS (**a**), FBS (**b**) and DMEM (**c**). Fluorescence intensity as measured by hyperspectral imaging of a control solution or solutions of ICG, Ag_2S dots or Ag_2S superdots in PBS (**d**), FBS (**e**) and DMEM (**f**).

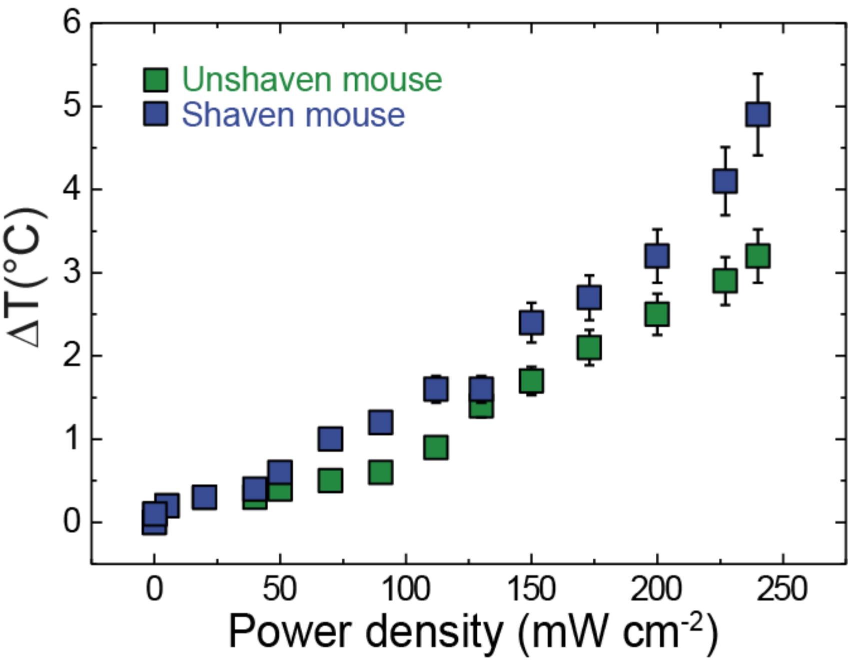

Supplementary Figure 16. *In vivo* laser-induced heating. Laser-induced temperature increment at the skin surface of a CD1 mouse for different 808 nm continuous wave illumination power densities (see Supplementary Methods for details). The maximum temperature increment observed was under 0.5 °C for the power density used for real-time *in vivo* imaging (50 mW cm^{-2}) with the Ag_2S superdots developed in this work. Error bars are given by the time fluctuations of the readout given by the thermal camera.

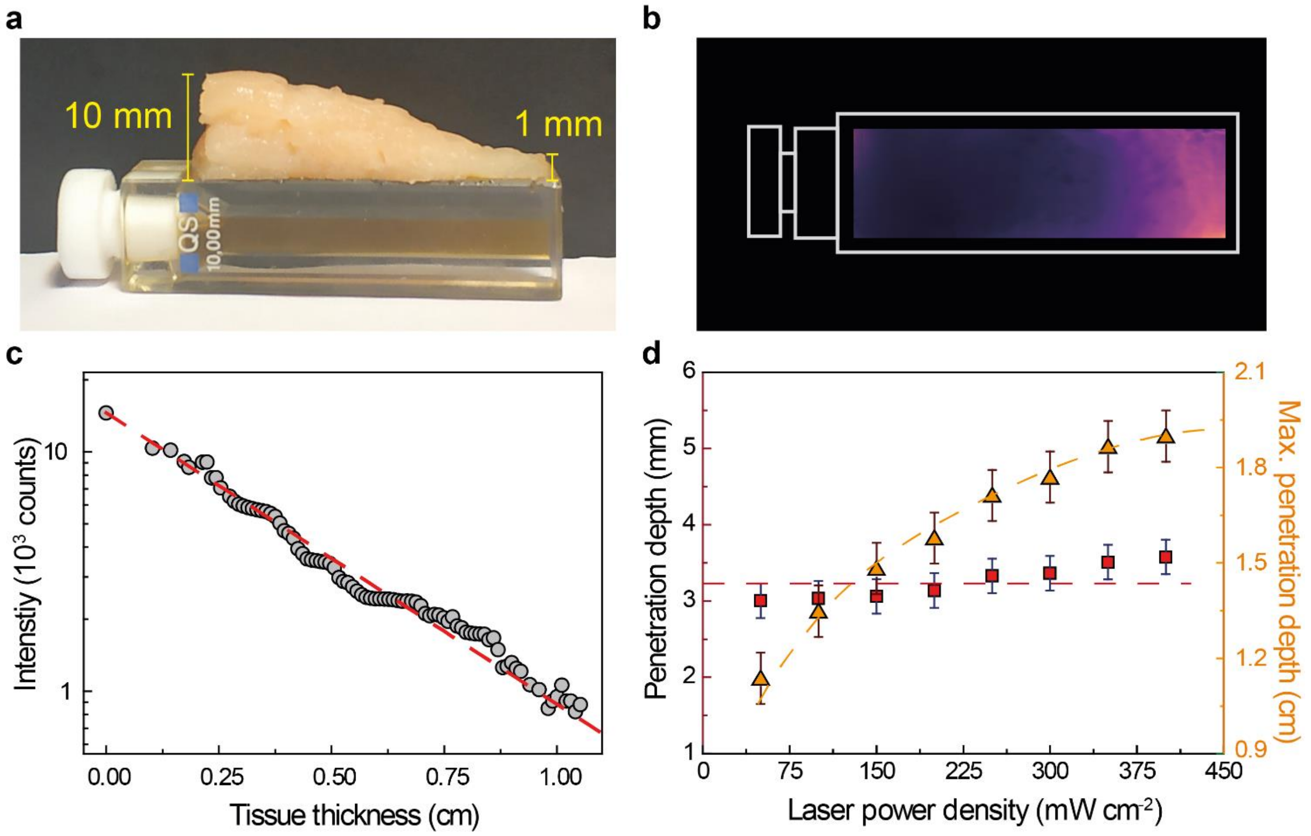

Supplementary Figure 17. Penetration of Ag_2S superdot emission into tissues. a Optical image of a cuvette filled with Ag_2S superdots and with a tissue of variable thickness on top. **b** Fluorescence image obtained when illuminating the system with an 808 nm laser. **c** Fluorescence intensity at different tissue depths. **d** l_{trad}, and l_{max} penetration depths for different laser power densities. Error bars in **d** are calculated based on the intensity fluctuations of the infrared fluorescence camera used for the acquisition of fluorescence images.

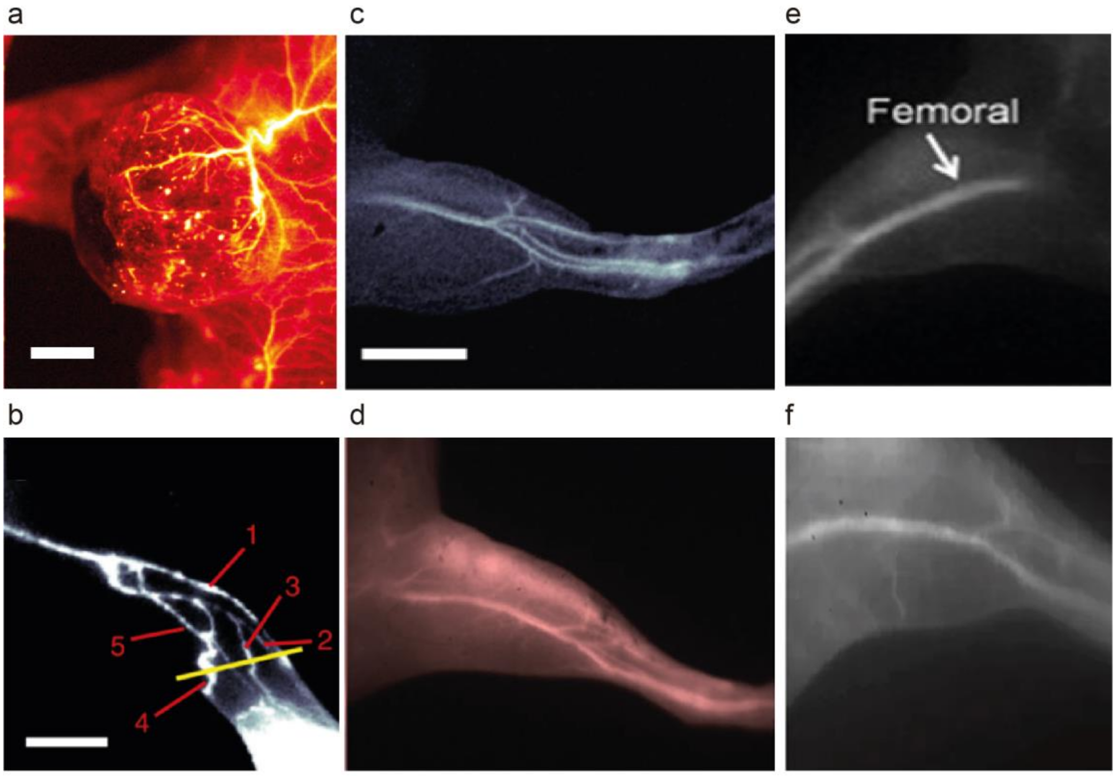

Supplementary Figure 18. Representative NIR-II *in vivo* fluorescence images of mouse vasculature. These were obtained by several research groups using different NIR-II probes and detection systems, summarized in Supplementary Table 3. **a**, **b**, **c**, **d**, **e** and **f** are reproduced with permission from references 4-9. Scale bars are 4 mm (**a**), 2.5 mm (**b**) and 6 mm (**c**).

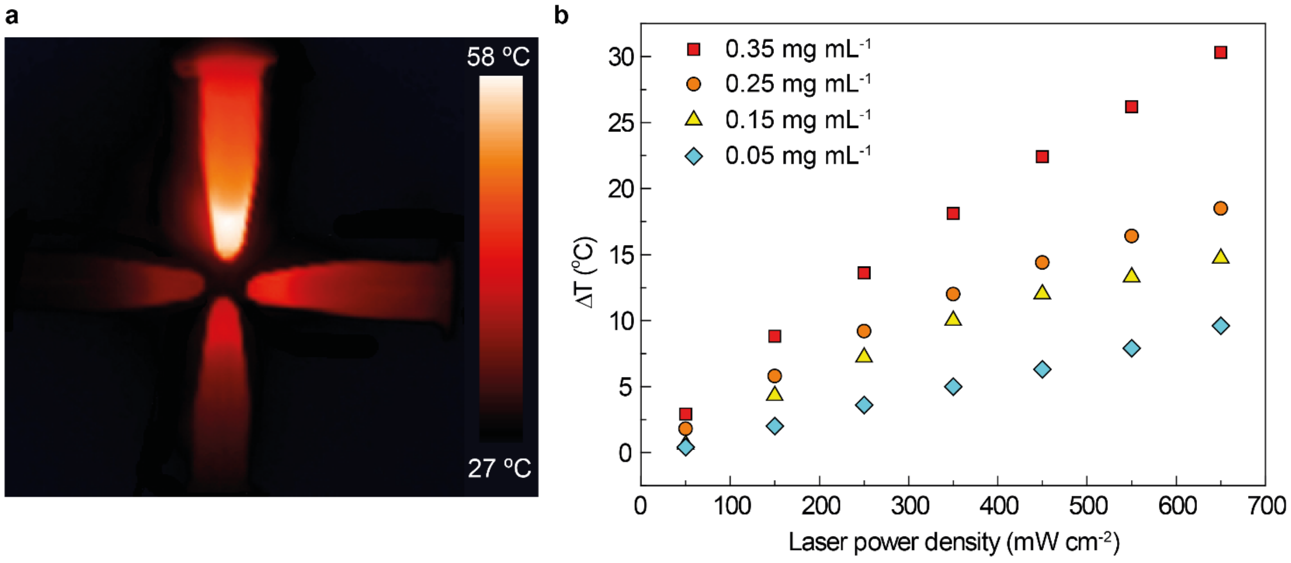

Supplementary Figure 19. Thermal loading in Ag_2S superdots. a Thermographic image of Eppendorf tubes containing aqueous dispersions of Ag_2S in different concentrations. **b** Heating efficiency curve obtained for the different dispersions of Ag_2S.

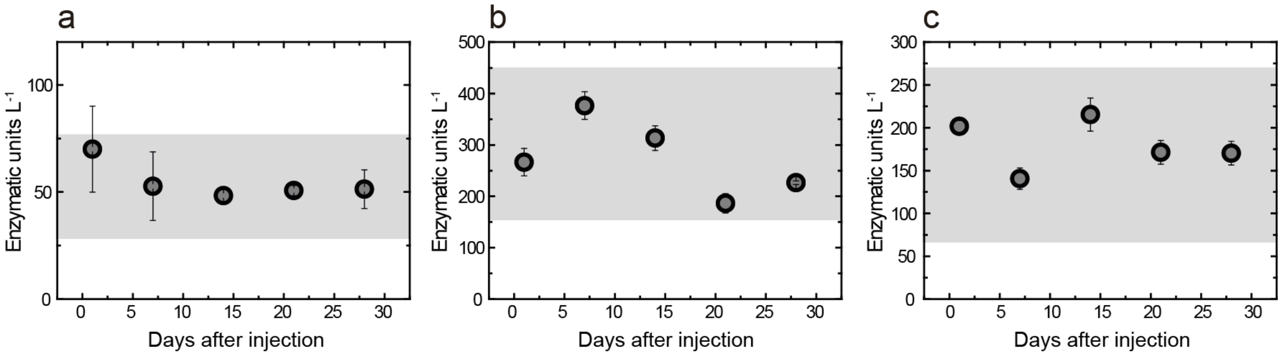

Supplementary Figure 20. Analysis of hepatic function. a-c Time evolution of ALT, ALP and AST enzyme contents after intravenous injection of Ag_2S superdots, respectively (n = 3 for all groups). The error bars correspond to the standard error of the mean and the grey areas indicate the reference ranges associated to healthy mice.

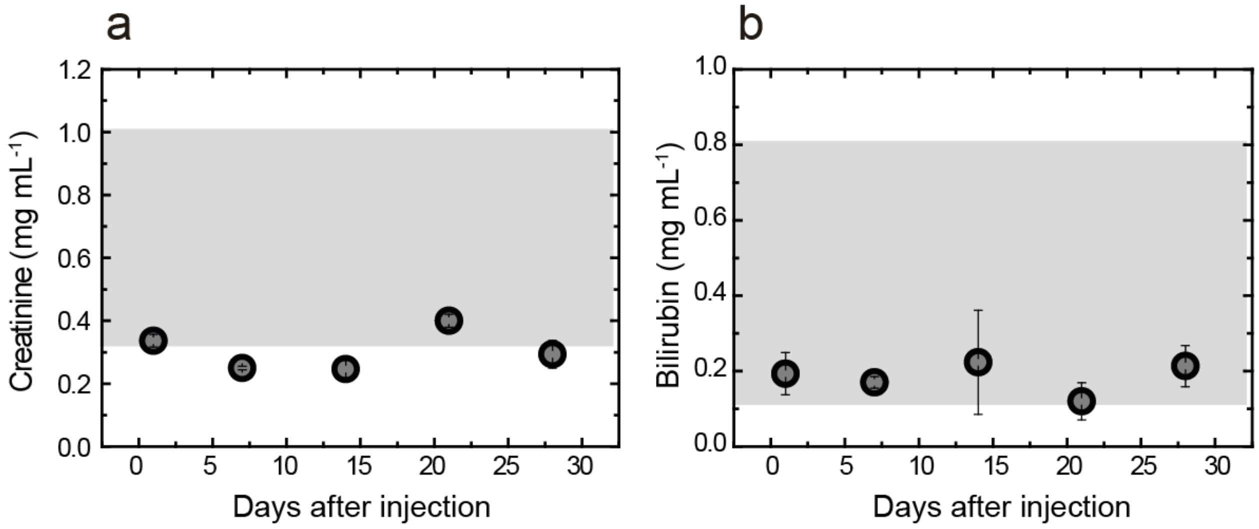

Supplementary Figure 21. Analysis of renal function and haemolysis. a-b Time-course of creatinine and total bilirubin concentration after intravenous injection of Ag_2S superdots, respectively (n = 3 for all groups). The error bars corresponding to standard error of the mean and the grey areas indicate the reference ranges associated to healthy mice.

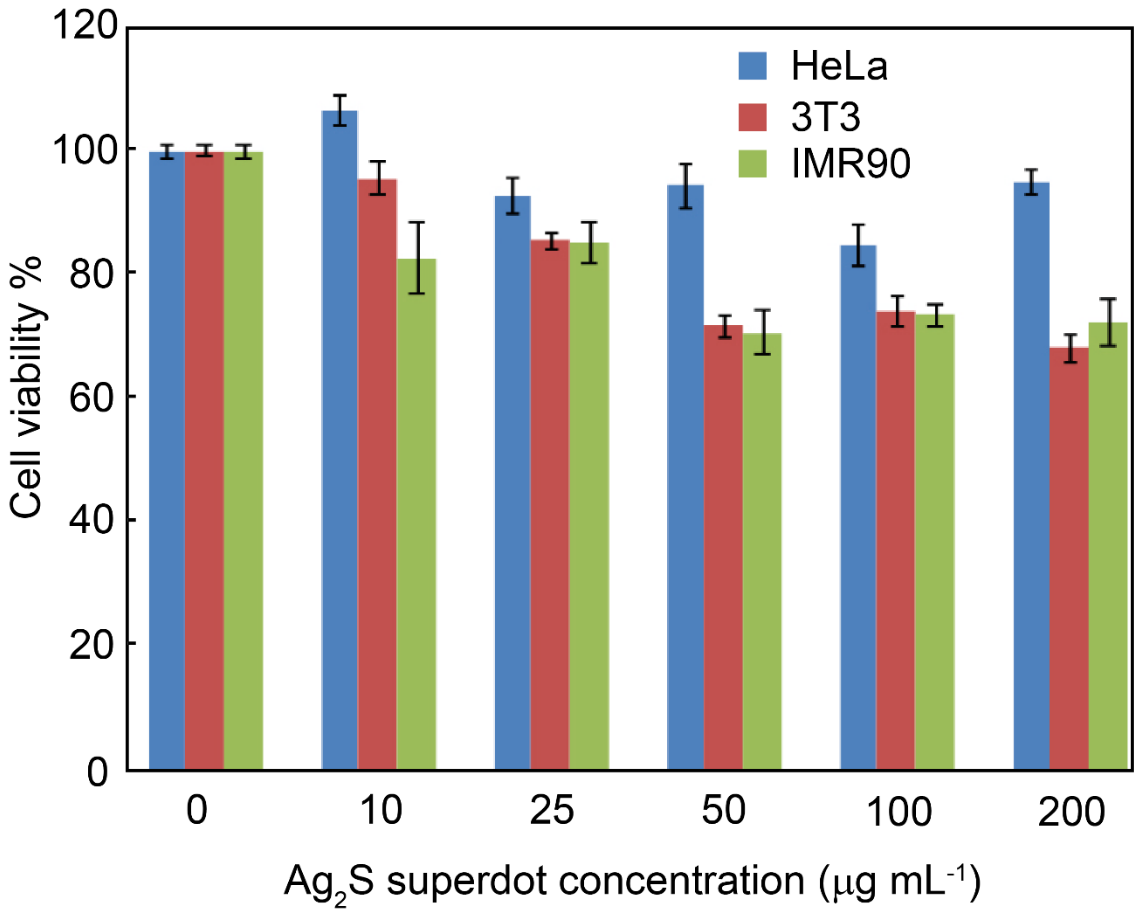

Supplementary Figure 22. Cytotoxicity. Cell viability of of HeLa, 3T3 and IMR90 cell after 48 h of incubation with different concentration of Ag_2S superdots in the cell medium as determined by MTT assay. Experiments were carried out four times and data were represented as the mean with the error bars being the standard deviation of the error.

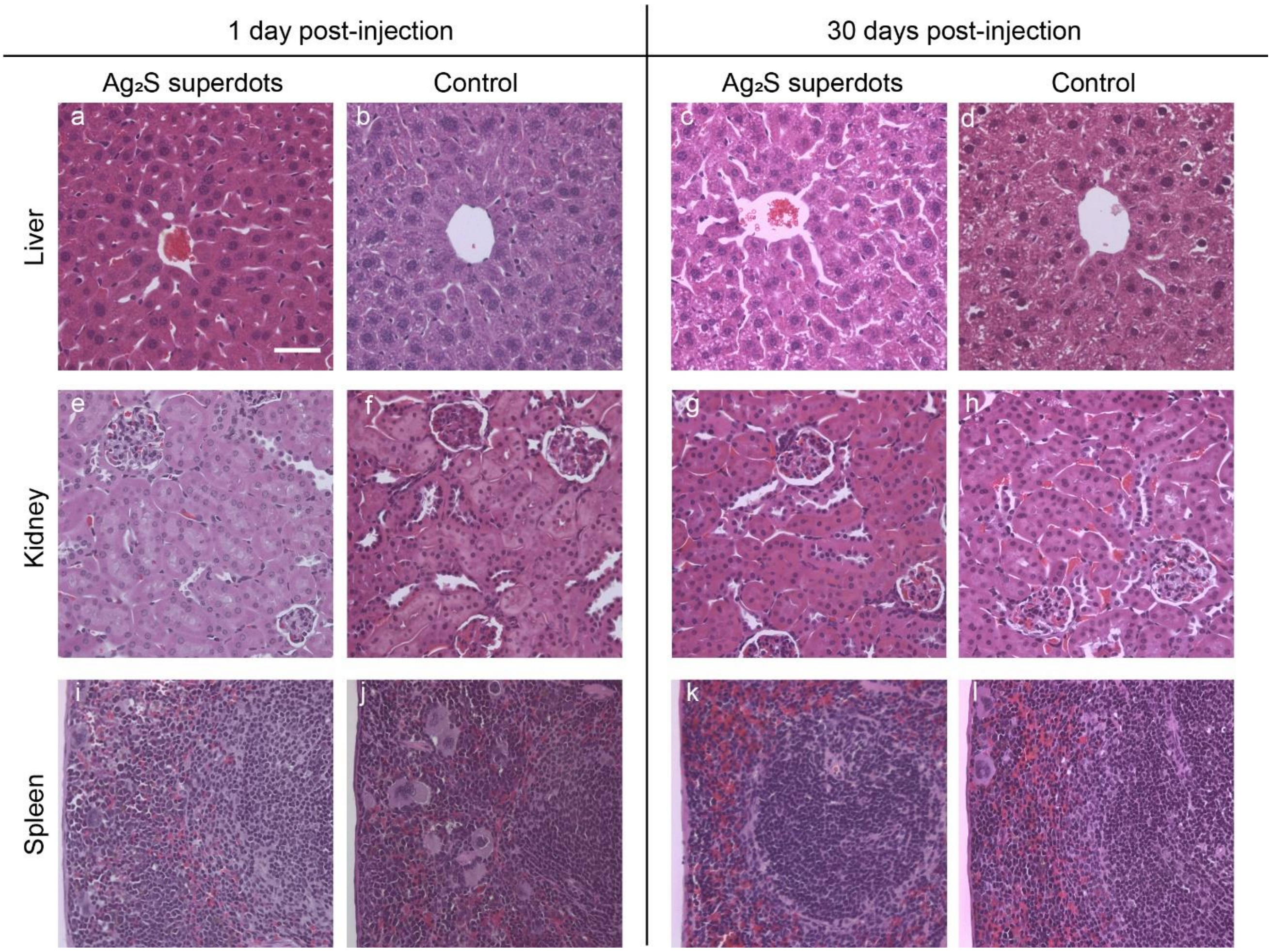

Supplementary Figure 23. Histological analysis. Histological images of liver (**a-d**), kidney (**e-h**) and spleen (**i-l**) of animals injected with Ag_2S superdots (**a, c, e, g, i, k**) and control animals (**b, d, f, h, j, l**) 1 and 30 days post-injection. All images were stained with hematoxylin-eosin staining method (bar size: 50 µm). The liver of animals injected with Ag_2S superdots showed a normal architectural hepatic structure and no abnormal hepatocyte changes were observed. No lesions were found in portal canals, central veins or hepatic sinusoids, and their morphological properties were similar to the control cases. No histological differences were observed in the liver at day 1 or at 30 days after injection of Ag_2S superdots. Kidney cytoarchitecture of animals injected with superdots was similar at day 1 and day 30 and no structural glomerular structure and proximal or distal tubes lesions were observed with respect to control kidneys. No changes acute (1 day post-injection) or chronic (30 days post-injection) were observed in spleen follicle and red pulp parenchyma. No necrotic or haemorrhagic areas were visualized in any of the tissues studied.

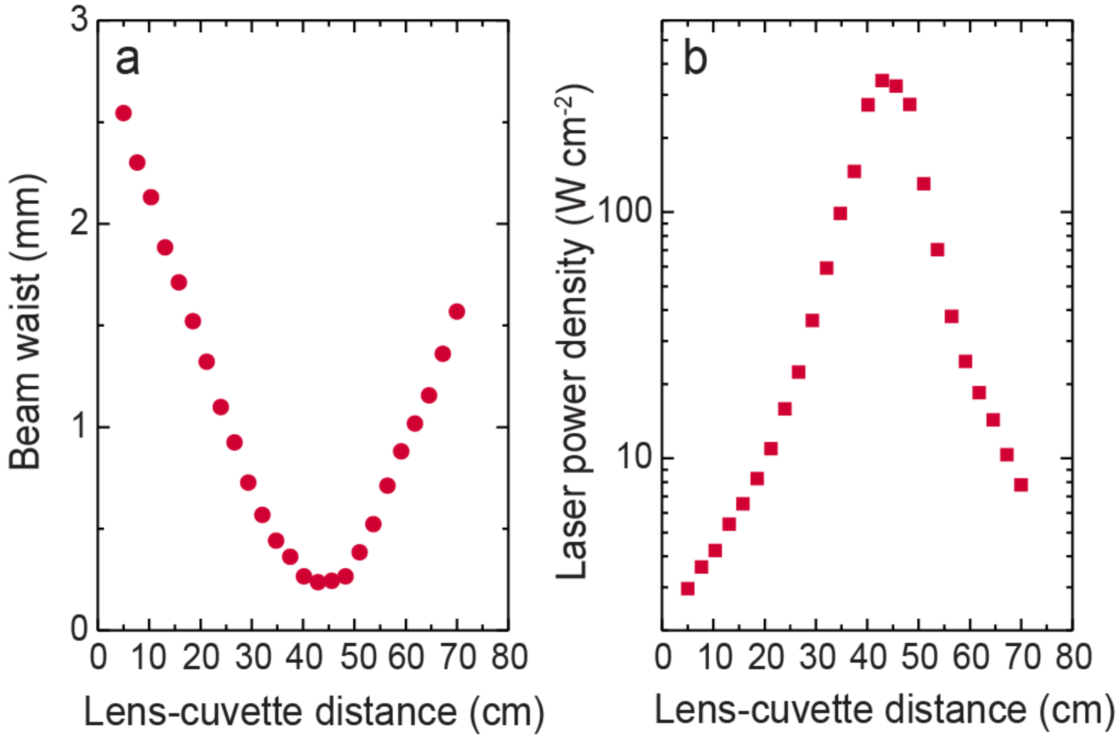

Supplementary Figure 24. Power density control during ultrafast irradiation. a Variation of the beam waist of the femtosecond laser pulse as a function of the lens-to-cuvette distance. **b**Variation of the power density of the femtosecond laser as a function of the lens-to-cuvette distance. The average laser power of the ultrafast irradiating laser was set to 0.6 W.

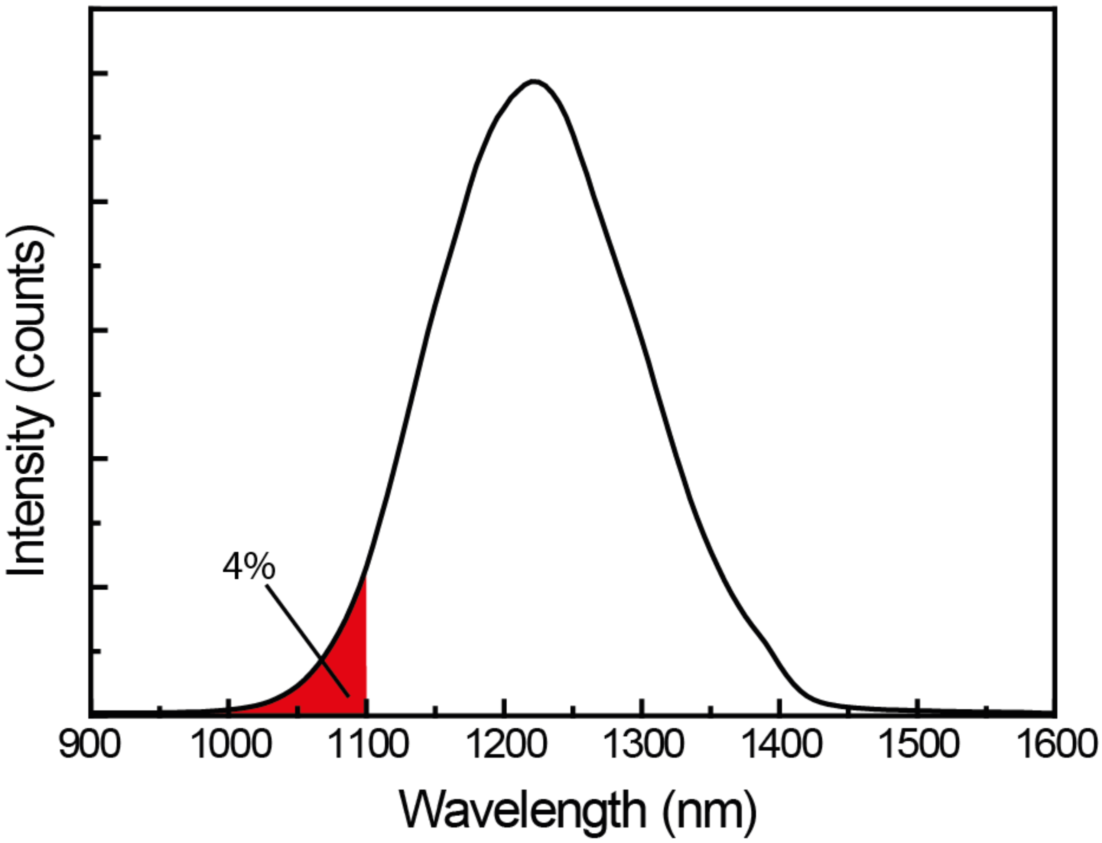

Supplementary Fig. 25. Short wavelength contribution to overall intensity. Schematic diagram of the contribution of photons under 1100 nm (shadowed in red) to the overall intensity emitted by Ag_2S superdots. The net contribution of photons at wavelengths below 1100 nm to the overall intensity is below 5 %.

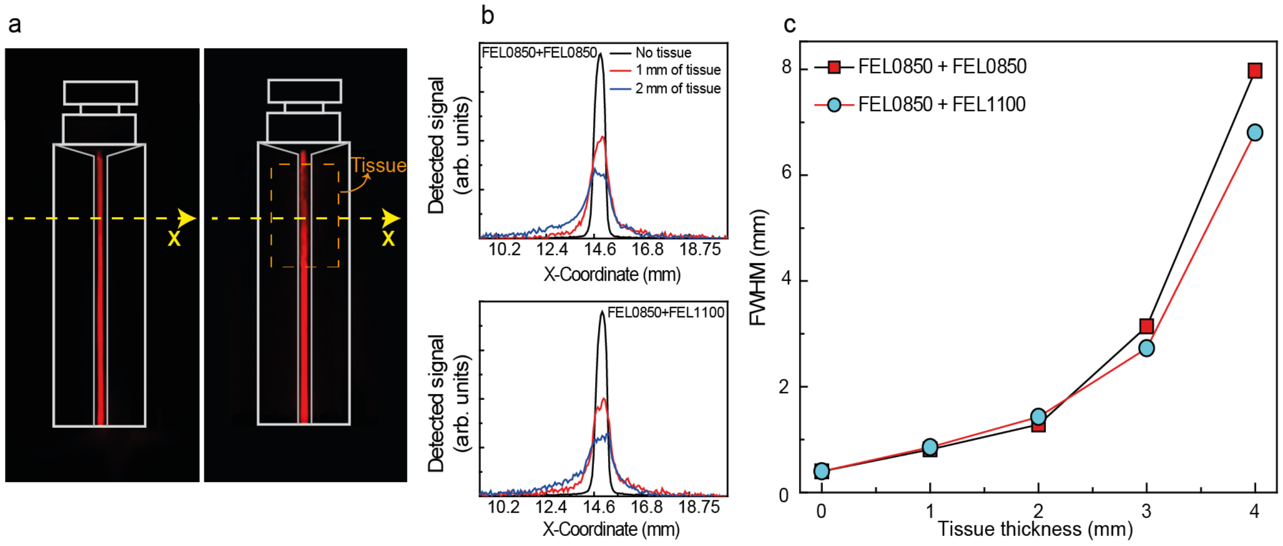

Supplementary Figure 26. Effect of a 1100 nm longpass filter on the spatial resolution of fluorescence images. a Fluorescence images of a cuvette filled with a dispersion of Ag_2S superdots illuminated by an 808 nm laser. The dashed rectangle on the right corresponds to the region where a piece of tissue was placed on top of the cuvette. **b** X-profiles of fluorescence intensity after passing through different depths of tissue. **c** Comparison of FWHM values as obtained for different combinations of longpass filters.

Supplementary Table 1. Photoluminescence properties of NIR-II probes

NIR-II Probe	ε @800 nm [$M^{-1}cm^{-1}$]	NIR-II QY	NIR-II Brightness (= ε QY)	NIR-II lifetime
SWNTs (PBS)	8 10^6	5 10^{-4}	4 10^2	< 1 ns
ICG (PBS)	1.2 10^5	4.5 10^{-4}	5.4 10^1	< 1ns
LaF_3:Nd (PBS)	2 10^2	0.5	1 10^2	30 µs
Ag_2S dots ($CHCl_3$)	4.7 10^5	0.0013	1.1 10^3	70 ns
Ag_2S superdots ($CHCl_3$)	4.4 10^5	0.101	4.4 10^4	2.1 µs
Ag_2S dots (PBS)	4.6 10^5	0.0008	5.9 10^2	50 ns
Ag_2S superdots (PBS)	4.4 10^5	0.1	2.6 10^4	2 µs

Molar extinction coefficient at 800 nm, NIR-II fluorescence quantum yield (QY), NIR-II brightness and NIR-II fluorescence lifetime of the Ag_2S dots and superdots in both $CHCl_3$ and PBS. The data for other commonly used NIR-II fluorescent probes are also included.

Supplementary Table 2. **Reported properties of NIR-II probes and experimental conditions for *in vivo* imaging**

	Photoluminescence properties		Surface properties		*In vivo* imaging conditions			
**Fluorescent probe**	**Excitation λ (nm)**	**Emission λ (nm)**	**Coating / Functionalization**	**Z-potential (mV)**	**Concentration (mg mL^{-1})**	**V (μL)**	**Power density (W cm^{-2})**	**Ref.**
SWNTs	808	1150	PL–PEG	N/A	0.26	200	0.13	10
SWNTs	808	1200	DSPE-mPEG	N/A	500 nM	200	0.14	11
$NaYF_4$:Yb^{3+}, Er^{3+} NPs	980	1525	HAS	-13.6	2	200	0.14	12
RE-doped core/ multishell	800	1525	DSPE-PEG2000-COOH	N/A	5–100 nM	200	0.2	13
PbS/CdS/ ZnS QDs	808	1270	MPA	-24.2	0.04	100	0.10	14
IR-1061	808	900-1300	DSPE-mPEG	N/A	0.27	200	0.14	15
Ag_2S dots	808	1100	DOX@PEG	N/A	1.5	200	0.45	16
Ag_2S dots	808	1200	PEG	N/A	1	50	0.126	8
Ag_2S dots	808	1200	6PEG	N/A	1.34	200	0.14	17
Ag_2S dots	808	1200	PEG	N/A	1.5	200	0.14	18
Ag_2S superdots	808	1220	PEG	-30 mV	0.15	100	0.05	This work

Supplementary Table 3. NIR-II imaging conditions

Probe	Injected dose	P_{laser} (mW cm^{-2})	Camera specifications				Ref.
			Model	Noise	Pixel size (μm^2)	Operating T (°C)	
p-FE	200 µL of an OD 6.5 (at 808 nm) solution	70	Princeton Instruments 2D OMA-V, USA	5000 e/p/sec	30x30	-100	6
$NaLnF_4$: Gd,Yb,Er,Ce	200 µL, 3 mg mL^{-1}	100	NIRvana, Princeton Instruments	10 e/p/sec	20x20	-80	4
Cyanine dye	200 µL, 1.25 nmol	150	NIRvana, Princeton Instruments	150 e/p/sec	20x20	-85	5
Cyanine dye	30-150 µg	150	Princeton Instruments	Un-specified	Un-specified	Un-specified	7
Ag_2S dots	50 µL, 1 mg mL^{-1}	45	Photonic Science, UK	40 e/sec	15x15	-25	8
SWNTs	200 µL, 0.1 mg mL^{-1}	140	LN cooled NIRvana, Princeton Instruments	10 e/p/sec	20x20	-190	9
Ag_2S dots	100 µL, 0.15 mg mL^{-1}	40	Xeva 320, Xenics	10000 e/sec	30x30	-40	This work

Experimental conditions regarding fluorophore concentration, optical excitation power density and camera specifications previously reported for the acquisition of NIR-II *in vivo* fluorescencence images of vasculature.

Supplementary References

1. Azzi L, El-Alfy M, Martel C, Labrie F. Gender differences in mouse skin morphology and specific effects of sex steroids and dehydroepiandrosterone. *J Invest Dermatol* **124**, 22-27 (2005).

2. SAFETEC, GUIA DE ANIMALES DE LABORATORIO I.Second edition. Rettenmaier Iberica JRS.

3. Moed S, Zaman MH. Towards better diagnostic tools for liver injury in low-income and middle-income countries. *BMJ Global Health* **4**, e001704 (2019).

4. Li Y, Zeng S, Hao J. Non-invasive optical guided tumor metastasis/vessel imaging by using lanthanide nanoprobe with enhanced down-shifting emission beyond 1500 nm. *ACS nano* **13**, 248-259 (2019).

5. Wang S, *et al.* Anti-quenching NIR-II molecular fluorophores for in vivo high-contrast imaging and pH sensing. *Nat Commun* **10**, 1-11 (2019).

6. Wan H, *et al.* A bright organic NIR-II nanofluorophore for three-dimensional imaging into biological tissues. *Nat Commun* **9**, 1171 (2018).

7. Zhu S, *et al.* Repurposing Cyanine NIR-I Dyes Accelerates Clinical Translation of Near-Infrared-II (NIR-II) Bioimaging. *Adv Mater* **30**, 1802546 (2018).

8. Li C, *et al.* In vivo real-time visualization of tissue blood flow and angiogenesis using Ag_2S quantum dots in the NIR-II window. *Biomaterials* **35**, 393-400 (2014).

9. Hong G, *et al.* Multifunctional in vivo vascular imaging using near-infrared II fluorescence. *Nat Med* **18**, 1841-1846 (2012).

10. Welsher K, *et al.* A route to brightly fluorescent carbon nanotubes for near-infrared imaging in mice. *Nat Nanotechnol* **4**, 773-780 (2009).

11. Welsher K, Sherlock SP, Dai H. Deep-tissue anatomical imaging of mice using carbon nanotube fluorophores in the second near-infrared window. *Proc Natl Acad Sci U S A* **108**, 8943-8948 (2011).

12. Naczynski DJ, *et al.* Rare-earth-doped biological composites as in vivo shortwave infrared reporters. *Nat Commun* **4**, 2199-2199 (2013).

13. Wang R, Li X, Zhou L, Zhang F. Epitaxial Seeded Growth of Rare-Earth Nanocrystals with Efficient 800 nm Near-Infrared to 1525 nm Short-Wavelength Infrared Downconversion Photoluminescence for In Vivo Bioimaging. *Angew Chem Int Ed* **53**, 12086-12090 (2014).

14. Benayas A, *et al.* PbS/CdS/ZnS Quantum Dots: A Multifunctional Platform for In Vivo Near-Infrared Low-Dose Fluorescence Imaging. *Adv Funct Mater* **25**, 6650-6659 (2015).

15. Tao Z, *et al.* Biological imaging using nanoparticles of small organic molecules with fluorescence emission at wavelengths longer than 1000 nm. *Angew Chem Int Ed* **52**, 13002-13006 (2013).

16. Hu F, Li C, Zhang Y, Wang M, Wu D, Wang Q. Real-time in vivo visualization of tumor therapy by a near-infrared-II Ag_2S quantum dot-based theranostic nanoplatform. *Nano Res* **8**, 1637-1647 (2015).

17. Hong G, *et al.* In Vivo Fluorescence Imaging with Ag_2S Quantum Dots in the Second Near-Infrared Region. *Angew Chem Int Ed* **51**, 9818-9821 (2012).

18. Zhang Y, *et al.* Biodistribution, pharmacokinetics and toxicology of Ag_2S near-infrared quantum dots in mice. *Biomaterials* **34**, 3639-3646 (2013).
