# Supplementary material for: Ultrafast photochemistry produces superbright short-wave infrared dots for low-dose in vivo imaging
Source: Nat Commun. 2020 Jun 10;11:2933. doi: 10.1038/s41467-020-16333-2 (PMC7286912; doi:10.1038/s41467-020-16333-2)
Supplement: Supplementary file 4 — Source Data [file 41467_2020_16333_MOESM4_ESM.zip › SUPPORTING/Pictures S23.zip!/Histological figure.pdf]

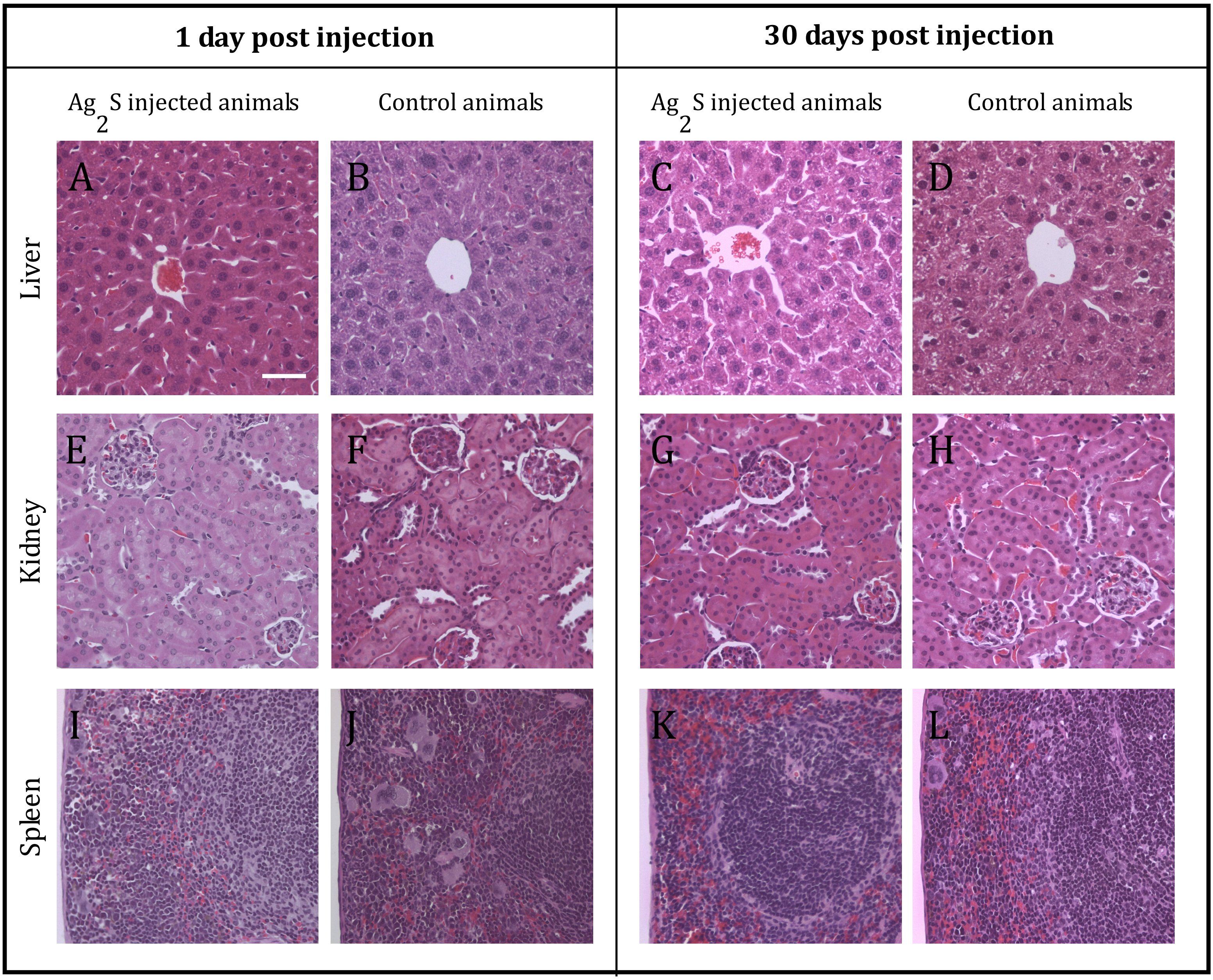
1 day post injection
30 days post injection
Ag2S injected animals
Control animals
Ag2S injected animals
Control animals
Liver
Kidney
Spleen
A
B
C
D
E
F
G
H
I
J
K
L
